# Comprehensive multiomics analysis identifies PYCARD as a key pyroptosis-related gene in osteoarthritis synovial macrophages

**DOI:** 10.3389/fimmu.2025.1558139

**Published:** 2025-03-24

**Authors:** Zihao Yao, Yuexin Li, Hanwen Mai, Zhuolun Wang, Haiyan Zhang, Daozhang Cai, Xiangjiang Wang

**Affiliations:** ^1^ Department of Joint Surgery, Center for Orthopaedic Surgery, The Third Affiliated Hospital of Southern Medical University, Guangzhou, China; ^2^ Department of Orthopedics, Orthopedic Hospital of Guangdong Province, Academy of Orthopedics·Guangdong Province, The Third Affiliated Hospital of Southern Medical University, Guangzhou, China; ^3^ The Third School of Clinical Medicine, Southern Medical University, Guangzhou, China; ^4^ Guangdong Provincial Key Laboratory of Bone and Joint Degeneration Diseases, Guangzhou, China; ^5^ Department of Orthopedics, The Affiliated Qingyuan Hospital (Qingyuan People’s Hospital), Guangzhou Medical University, Qingyuan, China

**Keywords:** pyroptosis, inflammation, PYCARD, osteoarthritis, synovial macrophages

## Abstract

**Background:**

Osteoarthritis (OA) is a chronic joint disease that significantly impairs quality of life. Synovitis plays a pivotal role in OA progression, and pyroptosis, a form of programmed cell death associated with innate immune inflammation, may contribute to the pathogenesis of OA synovitis. Nevertheless, the precise role of pyroptosis in OA pathogenesis remains poorly understood.

**Methods:**

We performed an analysis of bulk RNA sequencing data to examine the expression profiles of pyroptosis-related genes in the OA synovium. A LASSO-Cox regression model was employed to identify pivotal genes. Single-cell RNA sequencing data were used to validate the expression of these genes in specific synovial cell clusters. Differentially expressed genes (DEGs) in macrophages with high or low expression levels of core genes were subjected to enrichment analysis. A protein-protein interaction (PPI) network was constructed to identify hub genes, and potential therapeutic compounds were predicted. Consensus clustering analysis was performed to examine the correlations between hub genes and disease status. After identifying PYCARD as the core pyroptosis gene in OA macrophages, we assessed the expression levels of PYCARD in the OA synovium and validated the expression of PYCARD and its related core genes in M1 macrophages.

**Results:**

A total of twenty pyroptosis-related DEGs were identified, and six core genes were selected through LASSO regression. PYCARD was identified as the key pyroptosis gene in macrophages. Furthermore, 57 therapeutic compounds targeting these genes were predicted. Validation confirmed the upregulation of PYCARD in the OA synovium and M1 macrophages.

**Conclusion:**

PYCARD was identified as the core pyroptosis gene in OA macrophages, and 57 potential therapeutic compounds were identified. This study offers valuable insights into potential treatment targets for OA.

## 1 Introduction

Osteoarthritis (OA), the most prevalent joint disorder leading to disability, significantly reduces quality of life. Between 1990 and 2019, the global prevalence of OA increased by 48%. In 2019, OA was the 15th leading cause of years lived with disability worldwide, accounting for 2% of the total global YLD burden (1). As a complex disease with multifactorial origins, its development appears to be linked to factors such as mechanical overloading, trauma, inflammation, metabolic disturbances, and genetic susceptibility (2). Despite extensive research, the ambiguous pathogenesis of OA has hindered the identification of an effective cure. Current treatments focus primarily on pain management and joint lubrication, whereas knee replacement surgery remains the only viable solution for patients with advanced OA to regain some motor function (3). These findings underscore the importance of uncovering the underlying mechanisms of OA to advance prevention and treatment strategies.

Pathological changes in cartilage, subchondral bone, ligaments, menisci, fat pads, and synovium illustrate the systemic nature of OA as a disease impacting the entire joint (4). Notably, synovial abnormalities emerge at the early stages of OA, often preceding observable cartilage degradation, and the severity of synovitis is closely tied to disease progression (5). Synovial macrophages, the primary immune cells in this tissue, have been increasingly recognized for their critical role in OA pathogenesis (6). M1 macrophages, also known as classically activated macrophages, possess pro-inflammatory and antimicrobial functions. These cells secrete a variety of pro-inflammatory cytokines and mediators. In contrast to the pro-inflammatory M1 macrophages, M2 macrophages (also called alternatively activated macrophages) are primarily known for their anti-inflammatory functions, contributing to tissue repair, resolution of inflammation, and wound healing (7). In OA, the activation of M1 macrophages is often a response to damage-associated molecular patterns caused by joint tissue degeneration. An imbalance, with a higher proportion of M1 macrophages compared to M2 macrophages, exacerbates inflammation and tissue destruction, driving the progression of the disease (8). Therefore, targeting synovial macrophages is a promising avenue for developing OA therapies.

Pyroptosis is a distinct form of inflammatory cell death, separating it from noninflammatory processes such as apoptosis. Pyroptosis occurs through three distinct pathways—canonical, noncanonical, and alternative—each characterized by specific molecular mechanisms. The process unfolds in two main stages: initiation and activation. In the initiation phase of the canonical pathway, pathogen-associated molecular patterns and damage-associated molecular patterns interact with toll-like receptors, stimulating the MAPK and NF-κB signaling pathways. This leads to the cytoplasmic synthesis of the NLRP3 inflammasome, which is driven by alterations in mitochondrial DNA and reactive oxygen species. During the activation phase, the NLRP3 inflammasome subsequently activates caspase-1 (9–11). The study of pyroptosis has demonstrated a marked increase, particularly in its association with OA (12). Pyroptosis impacts a variety of tissues and cells, including osteoarthritis cartilage, the extracellular matrix, subchondral bone, the synovium, and joint fluid, in various ways. Although numerous studies have investigated pyroptosis-related molecules in OA, there remains no clear consensus on which cells are primarily affected or which undergo pyroptosis first (12, 13).

This research focused on analyzing the expression patterns of pyroptosis-associated genes in OA synovial cell clusters and pinpointing core genes and potential drugs related to pyroptosis. Key pyroptosis-related genes in the OA synovium were identified through extensive RNA sequencing data analysis. Single-cell RNA sequencing (scRNA-seq) data were analyzed to map the locations of pyroptosis-related genes to specific cell clusters. DEGs and enriched pathways were compared between cells with high and low expression levels of pyroptosis-related genes. Cytoscape software was used to identify hub genes among the cluster-specific DEGs. On the basis of the identified hub genes, candidate drugs for OA treatment were screened via the Drug–Gene Interaction Database (DGIdb). Among all pyroptosis-related genes, PYCARD was found to be upregulated in OA synovial macrophages. Hub genes of cells with high PYCARD expression were further validated to be upregulated in macrophages stimulated with LPS and IFNγ. Additionally, the candidate drug Butein was shown to alleviate macrophage pyroptosis, inflammatory polarization, and the expression of the hub gene ITGB2. Therefore, the pyroptosis-related gene PYCARD represents a potential biomarker for OA diagnosis and treatment.

## 2 Methods

### 2.1 Human synovial tissue

Samples of synovial tissue were collected from 10 patients with late-stage osteoarthritis who had knee replacement surgery and from 10 patients who had arthroscopy for trauma or joint problems. People suffering from hypertension, diabetes, high lipid levels, rheumatoid arthritis, other joint ailments, or a body mass index above 35 were excluded from the research. **Table 1** provides a summary of the participants’ demographic details, such as sex, age, and BMI. All participants provided informed consent, and the study received approval from the ethics committee of the Third Affiliated Hospital of Southern Medical University.

**Table 1:** Clinical characteristics of the study groups.

	NC (n=10)	OA (n=10)	P value
Sex			1
Female	5	6	
Male	5	4	
Age	62.8 (4.85)	64.5 (4.45)	0.402
BMI	26.25 (0.87)	25.43 (1.97)	0.269

### 2.2 Destabilization of the medial meniscus in an animal model

Ten-week-old male C57BL/6 mice were purchased from the Guangdong Experimental Animal Center, China. All animals were housed in pathogen-free cages with a temperature of 24 ± 5°C and a relative humidity of 40%. The study was conducted in strict adherence to the guidelines for animal welfare and ethical review of experimental animals (GB/T 35892-2018) and was approved by the Animal Care and Use Review Committee of Southern Medical University (License No: 2021-Ethical Review-053). All surgeries were performed under anesthesia using 0.3% pentobarbital sodium, and every effort was made to minimize suffering. For the medial meniscal instability OA model, mice were intraperitoneally injected with 0.3% pentobarbital sodium anesthetic. An incision was made along the medial collateral ligament, and the joint capsule was exposed. The femoral lateral condyle was exposed, and the medial meniscus was detached from its attachmeial plateau. After releasing the medial meniscus, the joint capsule and skin were sutured. Control mice underwent only skin incision and suturing. Mice were euthanized, and knee specimens were collected 8 weeks after surgery, as described previously (14).

### 2.3 Immunofluorescence staining

We deparaffinized and rehydrated mid-sagittal sections (4 μm thick) of paraffin-embedded clinical synovial samples. Mid-sagittal sections, 4 μm thick, of paraffin-embedded clinical synovial samples were deparaffinized and rehydrated. Antigen retrieval was achieved by placing the slides in Tris–EDTA buffer with a pH of 9.0 and applying microwave heating for 10 minutes. Following three washes with PBS, the slides were subjected to a 10-minute treatment with 3% hydrogen peroxide at room temperature. After blocking with 10% bovine serum (Solarbio, Beijing, China) for 1 hour at room temperature, the slides were incubated with primary antibodies at 4°C for 16 hours. The slides underwent a 1-hour incubation with the fluorescent dye at room temperature and were then mounted using DAPI mounting medium from Thermo Fisher Scientific. For the immunofluorescence assay, the reagents used included rabbit anti-PYCARD (Proteintech, 1:200, 10500-1-AP), mouse anti-CD68 (Proteintech, 1:200, 66231-2-Ig), mouse anti-F4/80 (Proteintech, 1:200, 27044-1-AP), and species-matched Alexa-488-or-594-labeled secondary antibodies (Life Technologies, Carlsbad, CA, USA).

### 2.4 Cell culture and cell transfection

THP-1 monocytes (American Type Culture Collection) were maintained in 1640 medium (Gibco, Carlsbad, CA, USA) supplemented with 10% fetal bovine serum, 100 units/mL penicillin, and 100 μg/mL streptomycin (Gibco). Differentiation into macrophages was induced by incubating monocytes for 24 hours with 150 nM phorbol 12-myristate 13-acetate (PMA, MCE, HY-18739), followed by an additional 24-hour incubation in 1640 medium. Macrophages were pre-treated with various concentrations of butein (5 μM and 10 μM) (MedChemExpress, #HY-16558) for 4 hours. To polarize the macrophages into M1 macrophages, the cells were treated with 20 ng/mL IFN-γ (R&D Systems, #285-IF) and 500 ng/mL LPS (Invitrogen, San Diego, CA, USA). Macrophage pyroptosis was induced by 200 ng/mL LPS for 5 hours, followed by ATP (5 mM) for 1 hour. PYCARD mRNA inhibition was achieved using small interfering RNA (siPYCARD). siPYCARD (80 nM) or siRNA negative control (siNC) (GenePharma, Suzhou, China) was transfected into macrophage cells using Lipofectamine 3000 (3 μL/mL) for 24 hours, following the manufacturer’s protocols.

### 2.5 qRT−PCR

M1 macrophages were cultured in 6-well plates, and total RNA was extracted using 1 mL of TRIzol reagent per well (Takara Bio Inc., Shiga, Japan). A reverse transcription kit from Vazyme Biotech in Nanjing, China, was employed to transcribe a 1 μg RNA sample into cDNA. Quantitative PCR (qPCR) tests were performed to measure the expression levels of PYCARD, TYROBP, FCER1G, AIF1, ITGB2, SPI1, CYBB, LAPTM5, C1QA, CD14, and HLA-DPA1 mRNAs in comparison to GAPDH mRNA. The experiments were conducted using a real-time PCR mix from Vazyme Biotech along with 2 × ChamQ SYBR qPCR Master Mix from the same company. The primers used for qPCR are listed in **Table 2**.

**Table 2:** Primers used in this study.

Gene	Primers	Sequences
PYCARD	Forward Sequence	AGCTCACCGCTAACGTGCTGC
	Reverse Sequence	GCTTGGCTGCCGACTGAGGAG
TYROBP	Forward Sequence	TGGTGCTGACAGTGCTCATTGC
	Reverse Sequence	CTGATAAGGCGACTCGGTCTCA
FCER1G	Forward Sequence	GTGCGAAAGGCAGCTATAACCAG
	Reverse Sequence	GGTGGTTTCTCATGCTTCAGAGT
AIF1	Forward Sequence	CCCTCCAAACTGGAAGGCTTCA
	Reverse Sequence	CTTTAGCTCTAGGTGAGTCTTGG
ITGB2	Forward Sequence	AGTCACCTACGACTCCTTCTGC
	Reverse Sequence	CAAACGACTGCTCCTGGATGCA
SPI1	Forward Sequence	GACACGGATCTATACCAACGCC
	Reverse Sequence	CCGTGAAGTTGTTCTCGGCGAA
CYBB	Forward Sequence	CTCTGAACTTGGAGACAGGCAAA
	Reverse Sequence	CACAGCGTGATGACAACTCCAG
LAPTM5	Forward Sequence	TTCCATCGCCTTCATCACTGTCC
	Reverse Sequence	CTCTTCTCCTCCACCGAGTTCA
C1QA	Forward Sequence	CGAGCACCAGACGGGAAGAAAG
	Reverse Sequence	AGGTTCCCCCTGGTCTCCTTTA
CD14	Forward Sequence	CTGGAACAGGTGCCTAAAGGAC
	Reverse Sequence	GTCCAGTGTCAGGTTATCCACC
HLA-DPA1	Forward Sequence	ATCCAGCGTTCCAACCACACTC
	Reverse Sequence	CGTTGAGCACTGGTGGGAAGAA

### 2.6 Western blot analysis

THP-1 cells were differentiated into macrophages by PMA stimulation and cultured in 6-well plates as previously described. Macrophages were lysed with 150 μL of radioimmunoprecipitation assay (RIPA) buffer containing protease and phosphatase inhibitors. Proteins were transferred to polyvinylidene difluoride (PVDF) membranes following electrophoresis. Membranes were incubated with primary antibodies diluted in 5% BSA in TBST for 14-16 hours at 4°C. The membranes were then incubated with secondary antibodies for 1 hour at room temperature. Target bands were visualized using FDbio-Dura ECL (FDbio Science, Hangzhou, China). The following antibodies were used for western blotting: anti-iNOS (Abcam, 1:1000, ab178945, USA), anti-ITGB2 (Proteintech, 1:1000, #10554-1-AP, China), anti-CD80 (Proteintech, 1:1000, #14292-1-AP, China), anti-GSDMD-N (Proteintech, 1:1000, #30695-1-AP, China), anti-NLRP3 (Abcam, 1:1000, ab263899, USA), and species-matched horseradish peroxidase-conjugated secondary antibodies (Jackson ImmunoResearch Laboratories, West Grove, PA, USA).

### 2.7 Data download

The datasets for synovial bulk RNA-seq include GSE55235 (10 healthy and 10 OA synovium samples), GSE55457 (10 healthy and 10 OA synovium samples), GSE82107 (7 healthy and 10 OA synovium samples), GSE55584 (6 OA synovium samples), and GSE89408 (28 healthy samples and 22 OA synovium samples), were retrieved from the Gene Expression Omnibus (GEO) database. Furthermore, the synovial scRNA-seq dataset GSE152805, consisting of 3 OA synovium samples, was obtained from GEO.

### 2.8 DEGs analysis

Differentially expressed genes (DEGs) were identified between the OA-related and control groups using the limma package, with the criteria of an FDR-adjusted p-value < 0.05 and a fold change (FC) > 1.5 or < 0.67 (15). Using the ggboxplot function from the ‘ggpubr’ R package, a boxplot was created to show pyroptosis-related genes in both normal and OA synovium groups (16). The ‘ggplot2’ R package’s ggplot function was employed to construct the volcano plot (17). The pheatmap function from the ‘pheatmap’ R package was used to create a heatmap.

### 2.9 Correlation analysis

The expression correlations of 20 DEGs related to pyroptosis were calculated for all samples and OA samples. The ‘Hmisc’ R package’s rcorr function was employed to conduct Pearson’s correlation analysis (18), and The results were displayed using the ‘corrplot’ package in R (19).

### 2.10 LASSO−Cox regression modeling

To develop a predictive model for osteoarthritis (OA) based on pyroptosis-related genes, LASSO regression analysis was conducted utilizing the “glmnet” R package (20). Given that OA is a nonfatal condition, survival times were standardized to a uniform value to mitigate their impact on the model. Healthy samples were designated as the baseline (0), while OA samples were assigned a status of 1 to represent the presence of OA. We standardized the survival times to a uniform value to allow the expression data to be processed in a consistent manner across all samples, without skewing the model due to the inherent differences in time-related factors in non-fatal diseases. The receiver operating characteristic (ROC) curve for each pyroptosis-related gene was generated using the roc function and subsequently visualized with the “pROC,” “survminer,” and “ggplot2” R packages (21, 22).

### 2.11 scRNA−seq quality check and batch effect correction

The raw gene expression data from GSE152805 were transformed into a Seurat object using the “Seurat” R package. Cells that had less than 200 expressed genes, more than 10,000 expressed genes, or more than 20% UMIs originating from the mitochondrial or ribosomal genome were removed. For the remaining cells, gene expression values were normalized on the basis of the total read count and mitochondrial percentage using the NormalizeData function, followed by standardization using the ScaleData function (23).

### 2.12 Dimensionality reduction and clustering

Principal components were calculated using the RunPCA function (23). To account for batch effects, Harmony was used to correct them (24). For visualization, the first 20 Harmony-aligned coordinates were plotted with the default settings of the RunTSNE function. Differential gene expression was assessed with the FindAllMarkers function, where the minimum percentage of expression was set to 0.25, and the threshold for log fold change was set to 0.25. Cell markers that are recognized by synovial cell clusters were identified and used for clustering analysis. The identified marker genes were then visualized using the dotplot function (25).

### 2.13 Differential gene expression and enrichment analyses

For the single-cell sequencing dataset, cells were divided into two clusters based on the median threshold of the target gene expression, and DEGs was assessed using the FindMarkers function in the Seurat R package. The following parameters were applied: logfc.threshold = 0.25 and p_val_adj = 0.05 (24). The biological functions of the identified DEGs across distinct clusters were evaluated using Gene Ontology (GO) enrichment analysis and Kyoto Encyclopedia of Genes and Genomes (KEGG) pathway analysis, which were conducted with the “clusterProfiler” R package (26). For the GO analysis, enriched biological processes, molecular functions, and cellular components were examined. A p-value adjusted to < 0.05 was considered statistically significant for the enrichment analysis, and the top 10 results from each category were selected for visualization.

### 2.14 Protein-protein interaction network construction and hub gene network identification

PYCARD-related DEGs and their associated genes were used to construct a PPI network using the STRING database. The “Multiple protein” mode was selected, with the organism set to “Homo sapiens” and the minimum interaction score set to a high confidence threshold of 0.700. The PPI network was visualized using Cytoscape (version 3.7.2) (27), and the cytoHubba plugin was employed to rank genes within the network based on their degree centrality values. The betweenness algorithm was applied within cytoHubba, while the Maximal Clique Centrality (MCC) algorithm was used for topology analysis and scoring the degree of interactions. Hub genes were defined as those with the top 10 highest scores (28).

### 2.15 Prediction of drug–gene interactions

The key genes identified as potential pharmaceutical targets for OA treatment were submitted to the DGIdb (http://www.dgidb.org/) to identify potential existing drugs or small organic compounds. For each drug–gene interaction, the reliability of the interaction was assessed based on evidence from relevant drug databases, such as DrugBank. The top 30 drugs with the highest interaction scores were selected as candidate therapeutic options for OA. A Sankey diagram was created using an online tool (https://www.lddgo.net/chart/sankey-chart) to visualize the drug–gene interactions.

### 2.16 Merge and batch effect correction of bulk RNA−seq datasets

The merging and batch effect correction of bulk RNA sequencing datasets were conducted by normalizing the GEO datasets GSE55457, GSE55235, GSE55584, GSE82107, and GSE89408. Data merging was conducted using the R package inSilicoMerging, and batch effect correction was handled using the R package sva with method set as COMBAT (29).

### 2.17 Sample clustering on the basis of PYCARD and its related genes

The expression matrix of PYCARD and its related genes from the merged bulk RNA-seq dataset was used for sample clustering and visualization via the R package ConsensusClusterPlus. The maximum number of clusters was set to 10, with 10 subsamples, a sampling proportion of 0.8, and the Pearson distance as the metric (30). The highest cluster consensus was achieved when k = 2. Principal component analysis was performed on clusters C1 and C2 via the prcomp function from the R package stats, and the results were visualized via the ggplot2 function.

### 2.18 Estimation of immune cell infiltration

The xCell algorithm is a gene signature-based approach that assigns relative infiltration scores to 64 immune and stromal cell types via gene expression data obtained from microarray sequencing (31). In this study, we applied the xCell algorithm and quanTIseq to assess the specific infiltration of immune cells across different clusters.

### 2.19 Statistical analyses

Data are presented as the mean ± standard deviation (SD). For comparisons between two groups, the Shapiro-Wilk test was performed to assess the normality of the data, followed by an unpaired Student’s t-test if the data met parametric assumptions. P-values less than 0.05 were considered statistically significant.

## 3 Results

### 3.1 Defining the expression patterns of pyroptosis-related genes in OA

**Figure 1:**
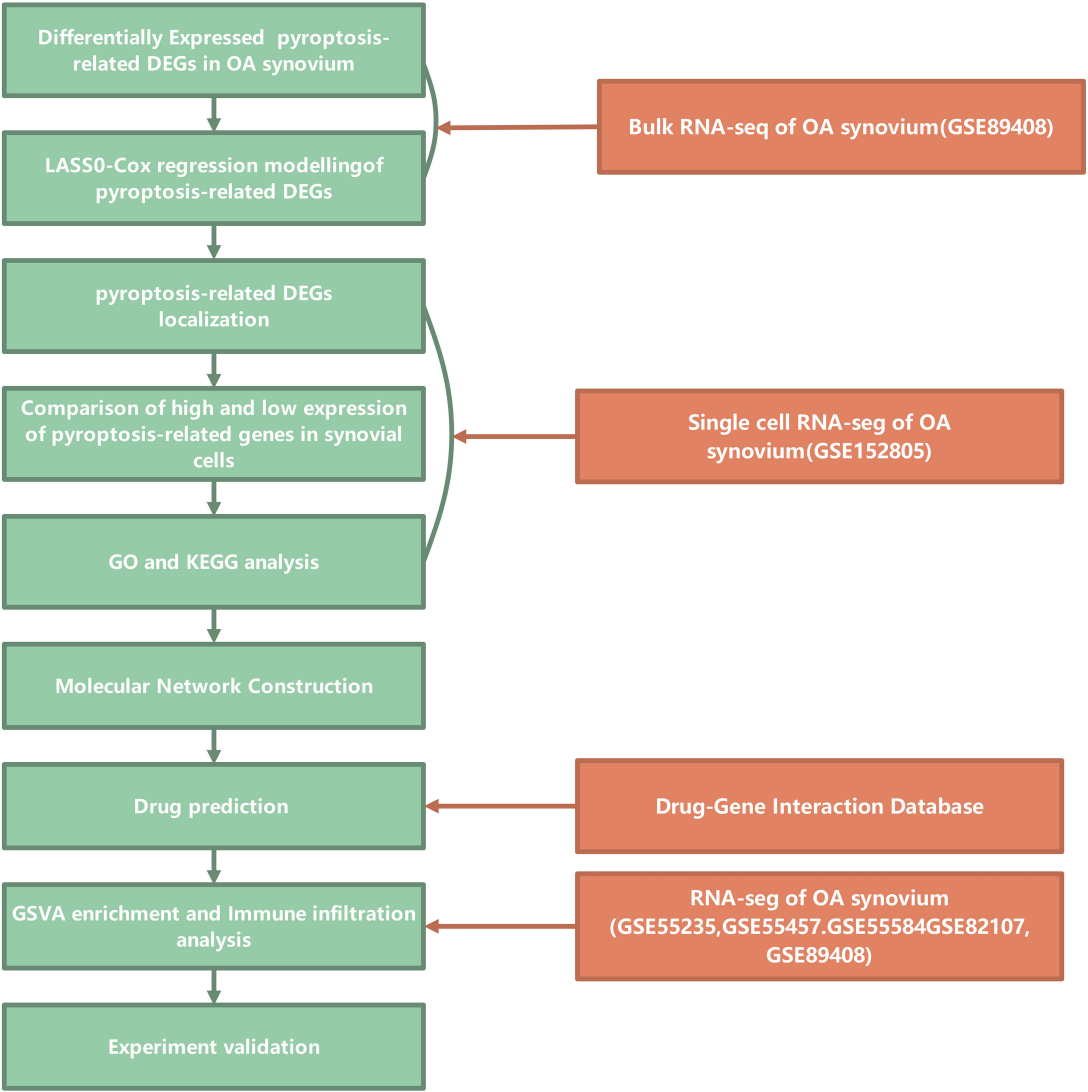
Study flowchart. Flowchart illustrating the bioinformatics analysis process for studying pyroptosis-related genes in osteoarthritis (OA) synovium. The analysis begins with the identification of differentially expressed pyroptosis-related DEGs (Differentially Expressed Genes) in OA synovium from bulk RNA sequencing data (GSE89408). LASSO-Cox regression modeling is applied to these DEGs to identify relevant genes. The localization of these genes in synovial cells is examined, followed by a comparison of high and low expression levels. Gene Ontology (GO) and Kyoto Encyclopedia of Genes and Genomes (KEGG) analyses are conducted to further investigate the functional roles of these genes. A molecular network construction is performed to explore the interactions between these genes. Drug predictions are made through the Drug-Gene Interaction Database, and the GSVA enrichment and immune infiltration analysis are carried out using additional RNA-seq datasets from GSE55235, GSE55457, GSE55584, GSE82107, and GSE89408. Finally, experimental validation is proposed to confirm the findings.

This study adhered to the workflow outlined in **Figure 1**. The bulk RNA-seq dataset GSE89408 was used to find genes associated with pyroptosis that show differential expression and to create a LASSO-Cox regression model for identifying key pyroptosis-related genes in the OA synovium. Subsequent analysis of the scRNA-seq dataset GSE152805 was conducted to pinpoint the locations of these core genes. DEGs and enriched pathways were then compared between cells with high and low expression levels of the core pyroptosis-related genes. Cytoscape software was utilized to identify hub genes among cluster-specific DEGs. Through the DGIdb database, these hub genes were screened to find potential drugs for OA treatment. A combined dataset from five GEO datasets (GSE55457, GSE55235, GSE55584, GSE82107, and GSE89408) was downloaded and analyzed for GSVA enrichment and immune infiltration of hub genes (32). These results highlighted a significant association between PYCARD and OA synovial macrophages. PYCARD expression was analyzed in clinical samples and in a destabilization of the medial meniscus animal model. The hub genes identified among the DEGs were further validated using qRT-PCR analysis in macrophages.

**Figure 2:**
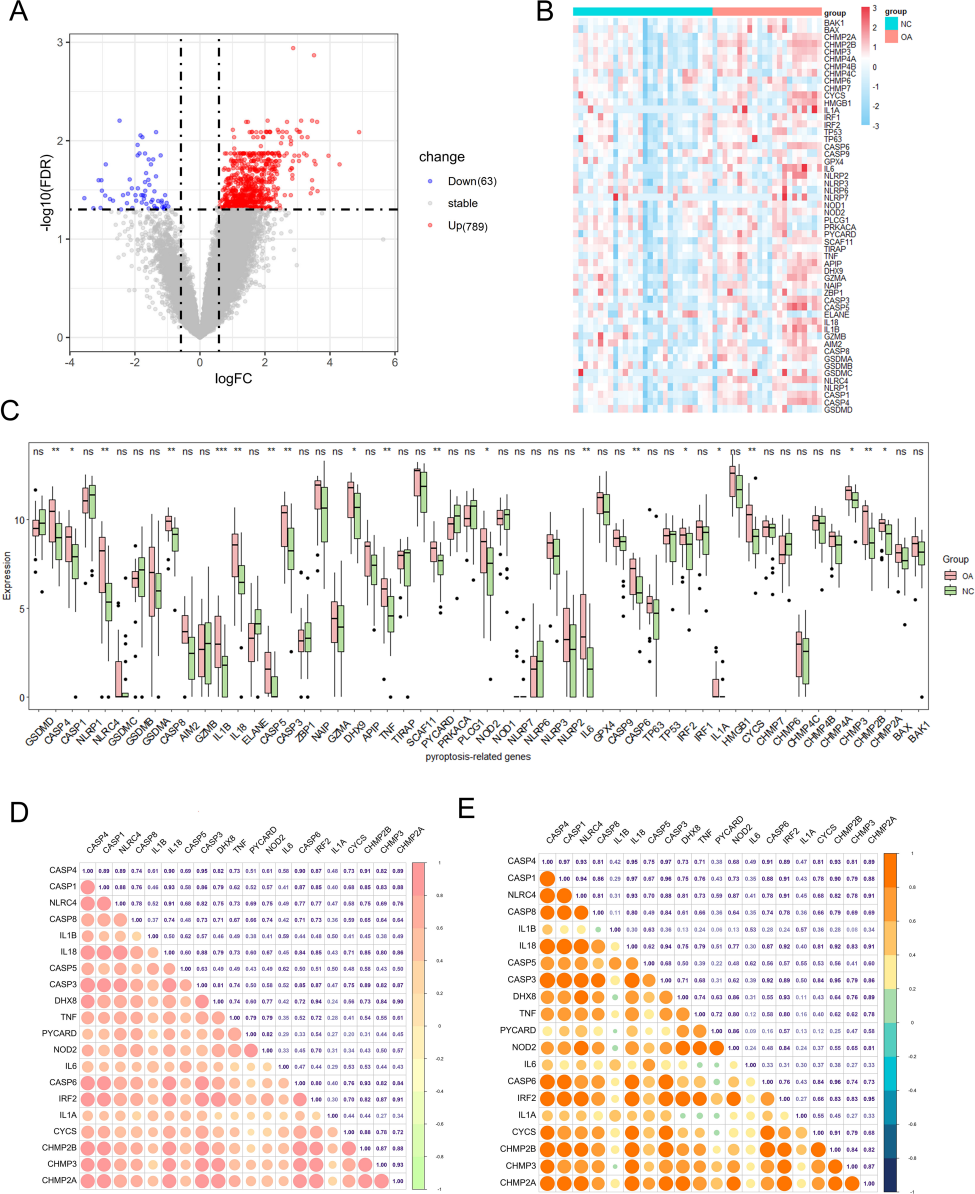
Expression of pyroptosis-related genes in OA. **(A)** Volcano plot of differentially expressed genes in OA vs. healthy synovium. Red and blue points represent genes significantly upregulated or downregulated in OA synovium (FDR < 0.05 and |fold change| > 1.5). The two vertical dashed lines represent ± log2(1.5) fold change, and the horizontal dashed line represents −log10(FDR-adjusted p-value cutoff of 0.05). **(B)** Heatmap showing the expression of key pyroptosis-related genes in OA and healthy synovium samples. **(C)** Box plots of key pyroptosis gene expression in OA and control groups. Red and green boxes indicate the expression levels of pyroptosis-related DEGs in OA and healthy synovium samples. *P < 0.05, **P < 0.01, ***P < 0.001, ns, not significant. **(D)** Correlation matrix of pyroptosis-related genes in the all samples. The size and color intensity of the circles represent the strength of the correlation, with positive correlations in red and negative correlations in green. **(E)** Correlation matrix of pyroptosis-related genes in the OA samples, showing similar visualization as **(D)**. The correlation coefficients are indicated within the circles.

Fifty-seven pyroptosis-related genes were identified from the literature (see **Supplementary Table S1**) (33). Our primary objective was to investigate alterations in the expression profiles and levels of pyroptosis-related genes in the OA synovium. Analysis of dataset GSE89408 from bulk RNA-seq identified 789 genes with higher expression and 63 genes with lower expression in OA synovium versus controls, revealing a unique expression pattern under OA conditions (**Figure 2A**). The expression levels of the 57 pyroptosis-related genes were further analyzed, and the results are presented as heatmaps for individual synovial samples and boxplots for all sample groups (**Figures 2B, C**). Of these genes, 20 (CASP4, CASP1, NLRC4, CASP8, IL1B, IL18, CASP5, CASP3, DHX8, TNF, PYCARD, NOD2, IL6, CASP6, IRF2, IL1A, CYCS, CHMP2B, CHMP3, and CHMP2A) were differentially expressed. The upregulation of these genes in the OA synovium suggests the involvement of pyroptosis in OA. We examined the expression correlation of the 20 pyroptosis-related DEGs in both the entire sample set and the OA sample subset from the GSE89408 dataset (**Figures 2D, E**). The results revealed that all DEGs were positively correlated. Notably, a significant positive correlation was observed between CASP3 and CASP4 expression in both the complete sample set and the OA sample subset.

### 3.2 The pyroptosis-related genes were used to construct an effective predictive model for OA

**Figure 3:**
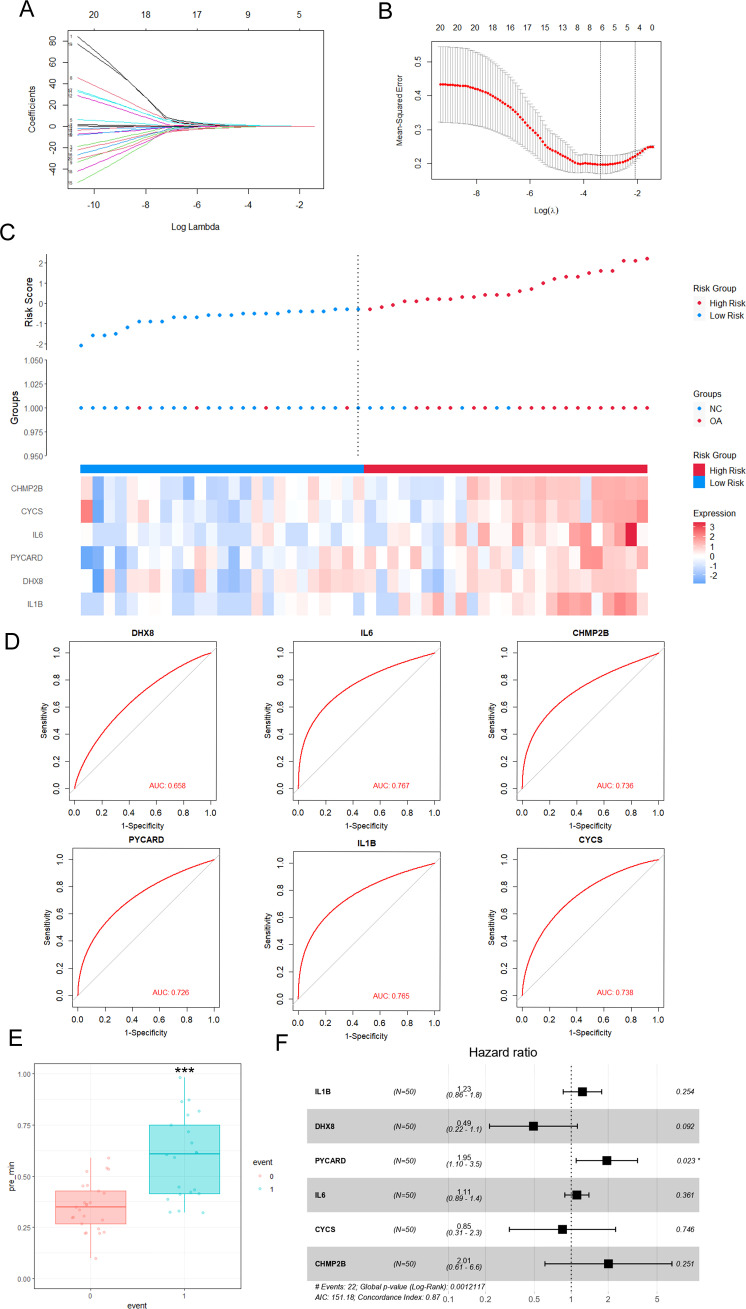
Effective predictive model of key pyroptosis-related genes for OA—LASSO-Cox regression analysis. **(A)** LASSO (Least Absolute Shrinkage and Selection Operator) coefficient plot showing the selection of 20 pyroptosis-related DEGs for the risk score model. The x-axis represents the log of the shrinkage parameter (log Lambda), and the y-axis shows the coefficients of the genes. **(B)** Cross-validation for tuning the parameter (Log(λ)) used in LASSO regression. The plot shows the mean squared error for each λ, with the optimal λ selected at the point where the error is minimized. **(C)** Heatmap of OA risk scores and expression of pyroptosis gene regulators. OA risk scores of the samples are arranged in ascending order. The top 50% of samples with the highest risk scores are classified as high-risk samples (red bars), while the remaining samples are classified as low-risk samples (blue bars). **(D)** Receiver operating characteristic (ROC) curves for selected genes (DHX8, IL6, CHMP2B, PYcard, IL1B, CYCS) in the risk score model, with AUC (Area Under the Curve) values displayed for each gene, demonstrating their diagnostic potential. **(E)** Boxplot showing the comparison of risk scores between normal and OA groups. A significant difference (***p < 0.001) is observed between the two groups. **(F)** Forest plot displaying the hazard ratios for each pyroptosis-related gene. The hazard ratios (HR) are derived from Cox regression analysis, showing the potential of these genes as prognostic markers for OA progression. Genes with statistically significant hazard ratios (p < 0.05) are indicated.

The GSE89408 dataset was analyzed using LASSO-Cox regression to identify important pyroptosis-related genes associated with OA progression and to develop an accurate OA predictive model. This approach enables precise predictive modeling and the identification of key variables (34). Using the LASSO regression algorithm, Using the optimal λ value, we incorporated six genes into the predictive model (**Figures 3A, B**). Six genes (IL1B, DHX8, PYCARD, IL6, CYCS, and CHMP2B) were selected for modeling: risk score = (0.20575382IL1B) + (−0.0935579DHX8) + (0.42108804PYCARD) + (0.1515762IL6) + (0.25919173CYCS) + (0.014998CHMP2B). In **Figure 3C**, the link between OA risk scores and the expression levels of the six genes is illustrated, highlighting their roles in the predictive model. The single-molecule ROC curves for IL1B, DHX8, PYCARD, IL6, CYCS, and CHMP2B yielded AUC values of 0.765, 0.658, 0.726, 0.767, 0.738, and 0.736, respectively. These findings suggest that IL1B, PYCARD, IL6, CYCS, and CHMP2B (AUC > 0.7) exhibit strong individual diagnostic value (**Figure 3D**). The OA group demonstrated a significantly higher risk score compared to the control group, as determined by the risk score formula (P < 0.001) (**Figure 3E**). Additionally, **Figure 3F** presents the hazard ratios for the six genes in the GSE89408 dataset. Five genes, including IL1B, DHX8, IL6, CYCS, and CHMP2B, were not significantly associated with OA risk, as their 95% confidence intervals crossed 1 (p > 0.05). In contrast, PYCARD was significantly associated with an increased risk (HR 1.95, 95% CI 1.10–3.5, p = 0.023). The global p-value of 0.0012 and a concordance index of 0.87 suggest a robust model, positioning PYCARD as a potential prognostic marker for OA risk.

### 3.3 scRNA-seq identified the specific localization of key pyroptosis-related genes

**Figure 4:**
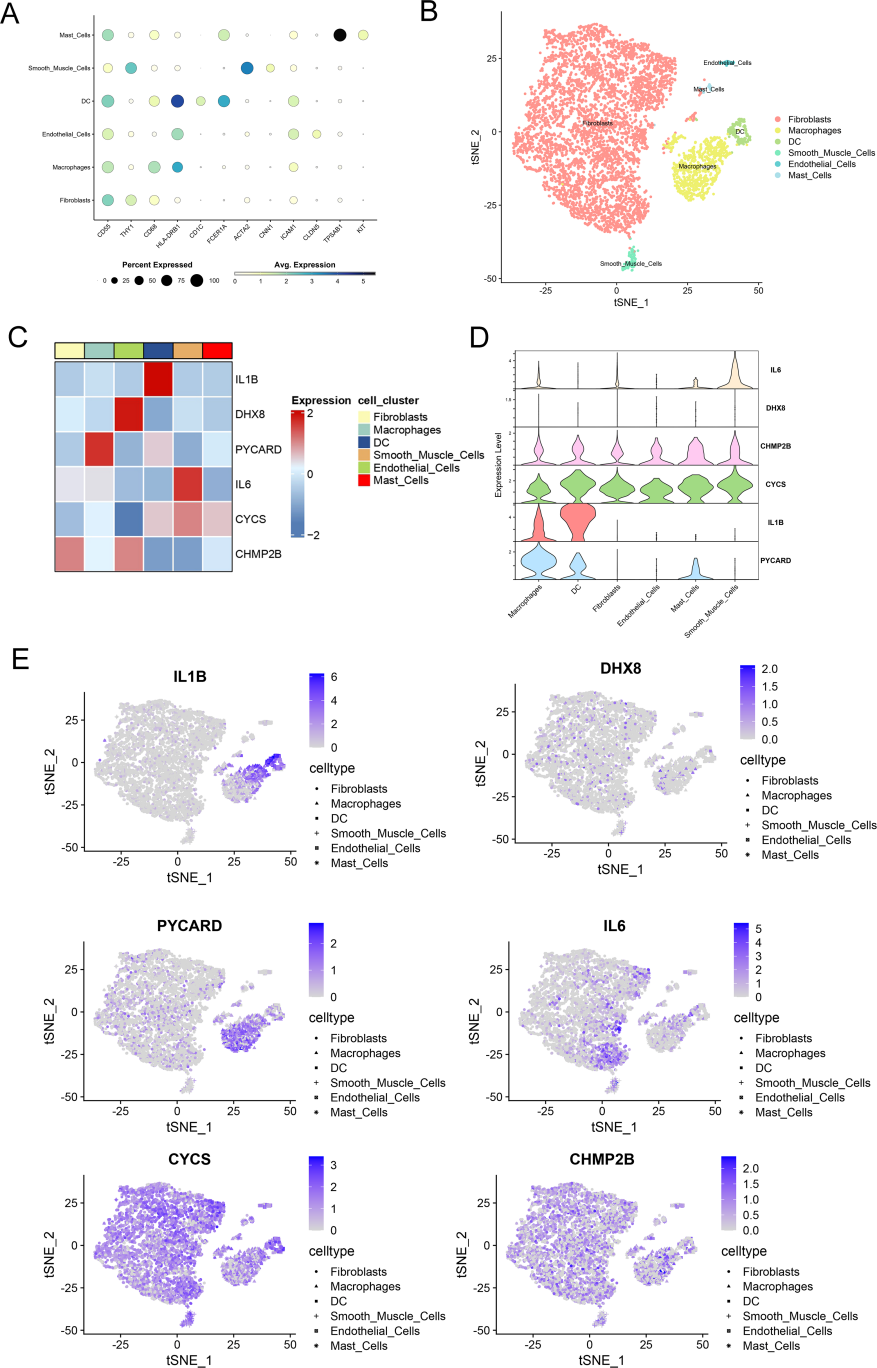
Single-cell sequencing demonstrates localization of key pyroptosis-related genes. **(A)** Dot plot showing the expression levels of pyroptosis-related genes across different cell clusters, including fibroblasts, macrophages, dendritic cells (DC), smooth muscle cells, endothelial cells, and mast cells. The size of each dot represents the percentage of cells expressing each gene, and the color indicates the average expression level. **(B)** t-SNE plot demonstrating the clustering of cells based on their gene expression profiles. Different cell types, including fibroblasts, macrophages, DC, smooth muscle cells, endothelial cells, and mast cells, are represented in different colors. **(C)** Heatmap depicting the expression levels of selected pyroptosis-related genes (IL1B, DHX8, PYcard, IL6, CYCS, CHMP2B) across various cell clusters. The color scale represents gene expression, with red indicating high expression and blue indicating low expression. **(D)** Violin plots of the expression of six key pyroptosis-related genes across synovial cell clusters. **(E)** Feature plots of key pyroptosis gene expression in each cell cluster. The color scale represents the expression levels of each gene, with darker shades indicating higher expression.

Using publicly available scRNA-seq data (GSE152805) from OA synovial samples, we studied the distribution of six genes related to pyroptosis. After performing data quality assessments and correcting for batch effects (**Supplementary Figures S1A, B, 2A, B**), a resolution of 1.2 was chosen based on the results of Clustree analysis (**Supplementary Figures S1C, D**). synovial cells were clustered into six distinct groups based on established cell markers (**Figures 4A, B**). PYCARD is predominantly expressed in macrophages in 3 synovial tissues (**Supplementary Figure S2C**). Heatmaps and feature plots were employed to visualize the expression levels of the six pyroptosis-related genes (**Figure 4C**). Our focus was primarily on two key clusters—fibroblasts and macrophages—as these are the principal effector cells involved in OA synovitis (35). Notably, CYCS and CHMP2B were predominantly expressed in fibroblasts, whereas CHMP2B, CYCS, IL1B, and PYCARD were highly expressed in macrophages, with PYCARD exhibiting the highest expression among all genes in macrophages (**Figures 4D, E**). Our previous analysis identified PYCARD as the most influential predictor of OA. Additionally, single-cell analysis revealed that PYCARD is most highly expressed in macrophages. These findings suggest that PYCARD plays a crucial role in regulating pyroptosis in macrophages within the OA synovium.

### 3.4 High PYCARD expression in macrophages is closely associated with pyroptosis

**Figure 5:**
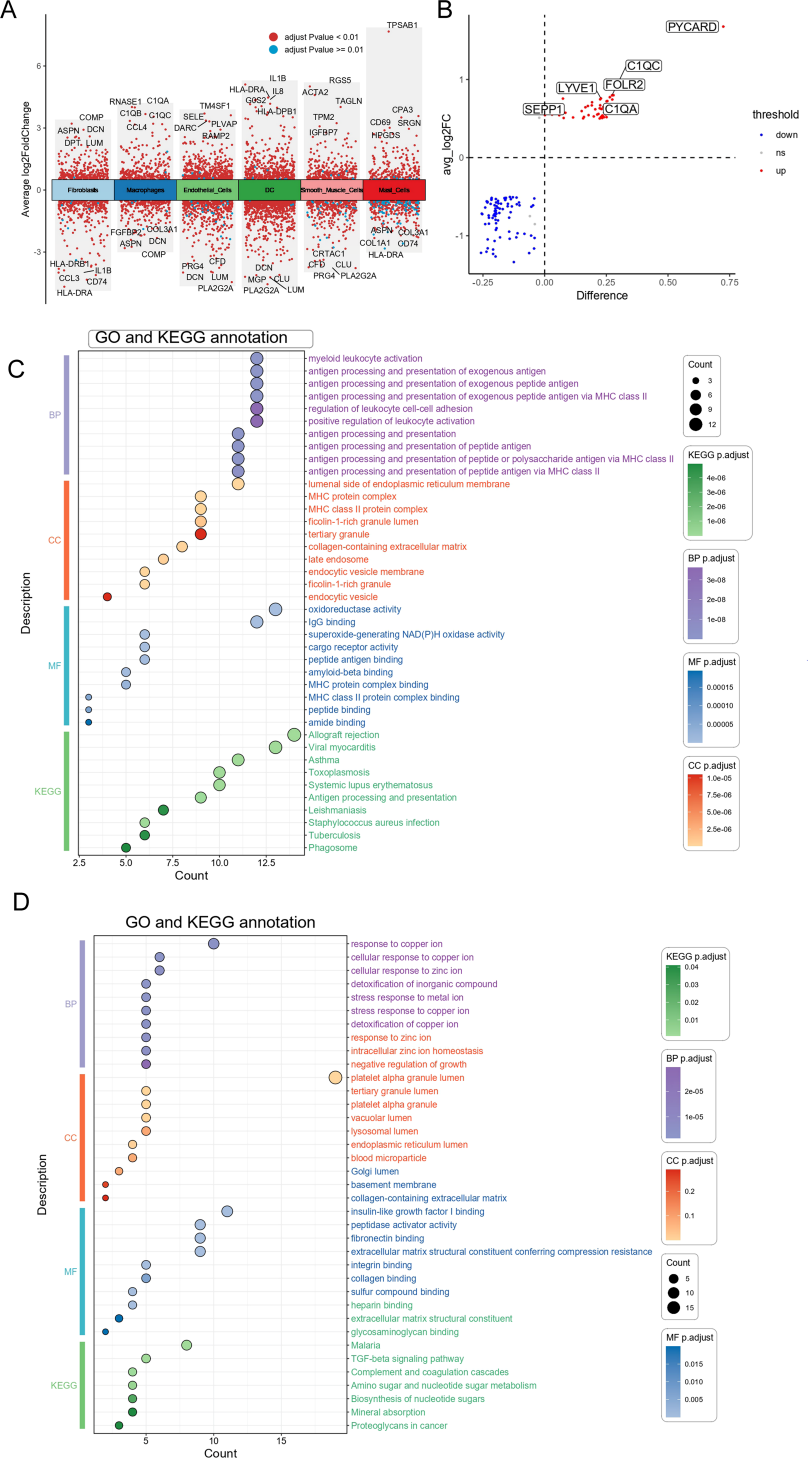
Differential genes and functional enrichment analysis of high vs. low PYCARD expression in macrophages. **(A)** Volcano plot showing differentially expressed genes (DEGs) between high vs. low PYCARD expression. Red dots represent genes with an adjusted p-value < 0.01, and blue dots represent those with an adjusted p-value > 0.01. The plot displays the average log2 fold change (x-axis) against the statistical significance (y-axis) of each gene. Genes are categorized into different cell types, including fibroblasts, macrophages, smooth muscle cells, endothelial cells, dendritic cells (DC), and mast cells. **(B)** Differential gene expression analysis of selected genes between high and low PYCARD-expressing macrophages, with genes marked as upregulated (red) and downregulated (blue). The difference in expression is plotted against the average log2 fold change. **(C)** Gene Ontology (GO) and Kyoto Encyclopedia of Genes and Genomes (KEGG) enrichment analysis of differentially expressed genes of High-PYCARD-related differential genes. The dot plot shows the top enriched terms across biological processes (BP), cellular components (CC), molecular functions (MF), and KEGG pathways, with the size of the dot representing the count and color representing the statistical significance (adjusted p-value). **(D)** Gene Ontology (GO) and Kyoto Encyclopedia of Genes and Genomes (KEGG) enrichment analysis of differentially expressed genes of Low-PYCARD-related differential genes.

We analyzed the single-cell dataset (GSE152805) to categorize cells into two groups based on PYCARD expression levels: a high-PYCARD group and a low-PYCARD group. A total of 128 DEGs were identified by comparing high- and low-PYCARD-expressing cells across distinct clusters, with notable genes such as C1QA, LYVE1, and SEPP1 specifically upregulated in macrophage populations (**Figures 5A, B**). **Figure 5C** illustrates that KEGG enrichment analysis identified significant enrichment of upregulated DEGs (high-PYCARD group) within immune-related processes, including “antigen processing and presentation,” “myeloid leukocyte activation,” and “phagosome,” all of which are closely associated with pyroptosis (36, 37). GO enrichment analysis of the upregulated DEGs (high-PYCARD group) identified that 7 out of the top 10 biological process, cellular component, and molecular function terms were associated with the major histocompatibility complex, which is closely linked to pyroptosis (38, 39). Additionally, **Figure 5D** shows that pathways of the downregulated DEGs (low-PYCARD group), such as “response to copper ion” and “TGF-beta signaling pathway,” suggest a connection between cellular stress responses and the regulation of pyroptosis. These findings highlight the crucial role of PYCARD in regulating pyroptosis and immune pathways at the single-cell level.

### 3.5 Construction of a PPI network using DEGs and prediction of drug–gene interactions

**Figure 6:**
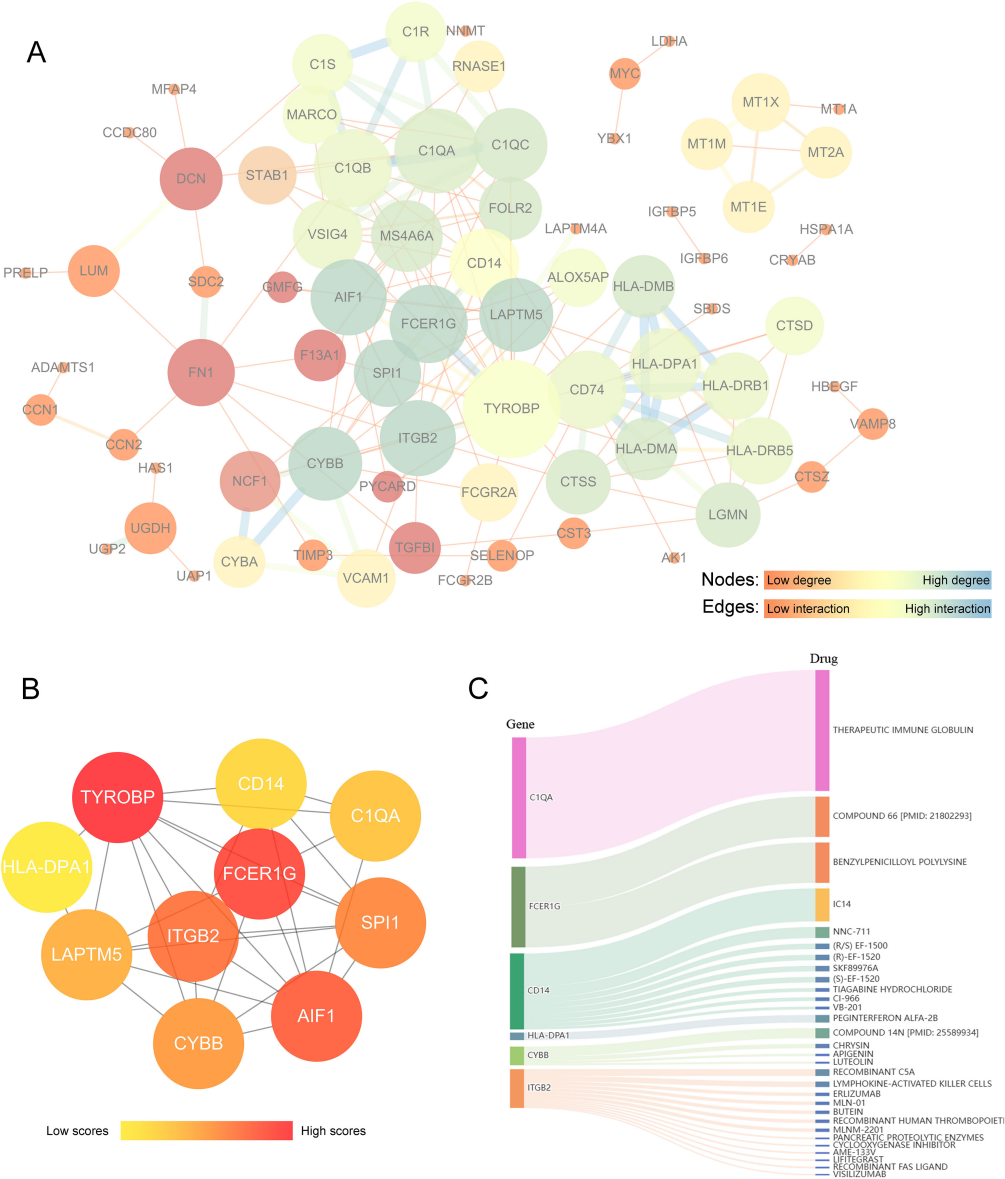
Analysis of key differential genes related to PYCARD and drug prediction. **(A)** Protein-protein interaction (PPI) network analysis of core differential genes associated with PYCARD. In this network, nodes represent proteins, and edges represent protein interactions. The color and size of the nodes indicate the degree centrality values, with darker blue representing proteins with high degree centrality, yellow indicating moderate degree, and brighter red reflecting lower degree. Larger nodes correspond to higher degree centrality. The thickness and color of the edges represent the strength or type of relationship between connected nodes, with darker blue and thicker lines indicating stronger interactions. **(B)** Hub gene analysis. The color intensity of the nodes indicates the level of interactions, with darker red nodes representing higher interaction levels. **(C)** Drug-gene interaction prediction of central genes. Ten key genes were targeted in the DGIdb database. The width of the flow lines indicates the strength of the interaction between the gene and the drug, with names of potential target drugs (e.g., Therapeutic Immune Globulin, Compound 66) listed among the top 30 drugs.

We utilized Cytoscape software to construct a PPI network for analyzing the interactions among the 128 DEGs across the two clusters (**Supplementary Table S2**), resulting in 74 nodes and 177 edges (**Figure 6A**). Using cytoHubba, the top 10 hub genes that had the highest scores were determined (**Figure 6B**). These hub genes were TYROBP, FCER1G, AIF1, ITGB2, SPI1, CYBB, LAPTM5, C1QA, CD14, and HLA-DPA1 (**Supplementary Table S3**). These genes are potential druggable targets for OA treatment. The DGIdb database identified 57 potential drugs or compounds targeting these genes for OA treatment (**Supplementary Table S4**). The top 30 drugs/compounds are displayed in **Figure 6C**. The visualization shows the genes on the left and the drugs on the right, with the interaction group score represented by the area size and the larger areas indicate higher scores. The drug THERAPEUTIC IMMUNE GLOBULIN, which targets C1QA, has the highest interaction score of 52. For FCER1G, two drugs—COMPOUND 66 [PMID: 21802293] and Benzylpenicilloyl Polylysine—each scored 17. Additionally, CD14 is targeted by 9 drugs, ITGB2 by 13 drugs, CYBB by 4 drugs, and HLA-DPA1 by 1 drug. However, no potential drugs have been identified for LAPTM5, AIF1, TYROBP, or SPI1.

### 3.6 PYCARD was strongly correlated with OA synovial macrophages

**Figure 7:**
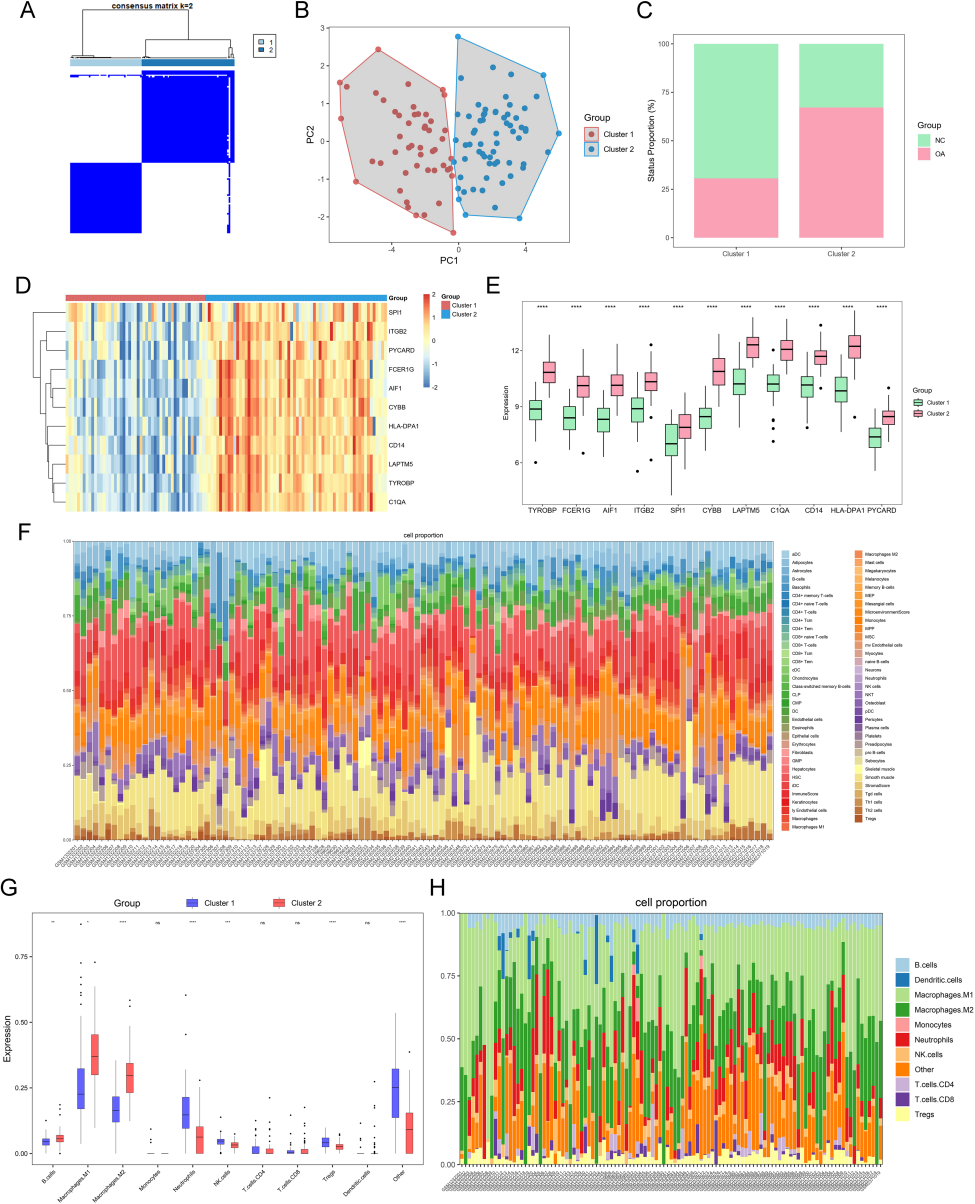
OA grouping and immune infiltration based on key differential genes related to PYCARD. **(A)** Heatmap of two synovial sample clusters identified using the ConsensusClusterPlus function, based on PYCARD and its related key differential genes. Cluster C1 contains 60 synovial samples, and cluster C2 contains 53 synovial samples. **(B)** Principal Component Analysis (PCA) plot displaying the separation of OA samples into two clusters, Cluster 1 (red) and Cluster 2 (blue). **(C)** Proportions of control and OA samples in clusters C1 and C2. **(D, E)** Expression heatmap and bar chart of PYCARD and its key related differential genes in clusters C1 and C2. **(F)** Stacked bar plot showing the cell composition of each sample. The cell types are color-coded, with the relative abundance of each cell type represented in the stacked bars. **(G)** Immune cell infiltration scores in the two pyroptosis-related clusters of major lineages such as Macrophages an B cells. **(H)** Stacked bar plot showing the major lineages cell composition of each sample. Statistical significance is indicated by asterisks (*p < 0.05, **p < 0.01, ***p < 0.001, ****p < 0.0001; ns, not significant).

We examined combined datasets from GSE55457, GSE55235, GSE55584, GSE82107, and GSE89408 to confirm the role of PYCARD and its associated DEGs in cell clusters of both control and OA synovium. The datasets were merged, and batch effect correction was performed (**Supplementary Figures S2A, B**). The 113 synovial samples were categorized based on the expression levels of PYCARD and its key DEGs, including TYROBP, FCER1G, AIF1, ITGB2, SPI1, CYBB, LAPTM5, C1QA, CD14, and HLA-DPA1. The samples were divided into two clusters according to the highest consensus values: C1 with 49 samples and C2 with 64 samples. (**Figure 7A**). Principal component analysis demonstrated distinct separation between clusters, indicating unique gene expression profiles (**Figure 7B**). The presence of OA synovium was observed in 15 (30.6%) of the C1 samples and 43 (67.1%) of the C2 samples, suggesting a stronger association of the C2 cluster with OA characteristics compared to C1 (**Figure 7C**). We analyzed the inter-cluster expression of PYCARD and its core DEGs and found significantly higher expression levels in cluster 2 (**Figures 7D, E**). We further performed immune infiltration analysis on 113 synovial transcriptomes using the xCell algorithm (**Figure 7F**). We analyzed the variations in immune cell infiltration between cluster 1 and cluster 2, as previously defined. Cluster 2 exhibited enhanced immune cell infiltration, particularly with increased macrophages of both M1 and M2 subtypes, while fibroblast levels remained consistent across both groups (**Figures 7G, H**, **Supplementary Figure S2D**). These findings highlight the potential role of PYCARD and its related DEGs in immune responses within the OA synovium and in macrophage phenotypic changes.

### 3.7 PYCARD was upregulated in the OA synovium

**Figure 8:**
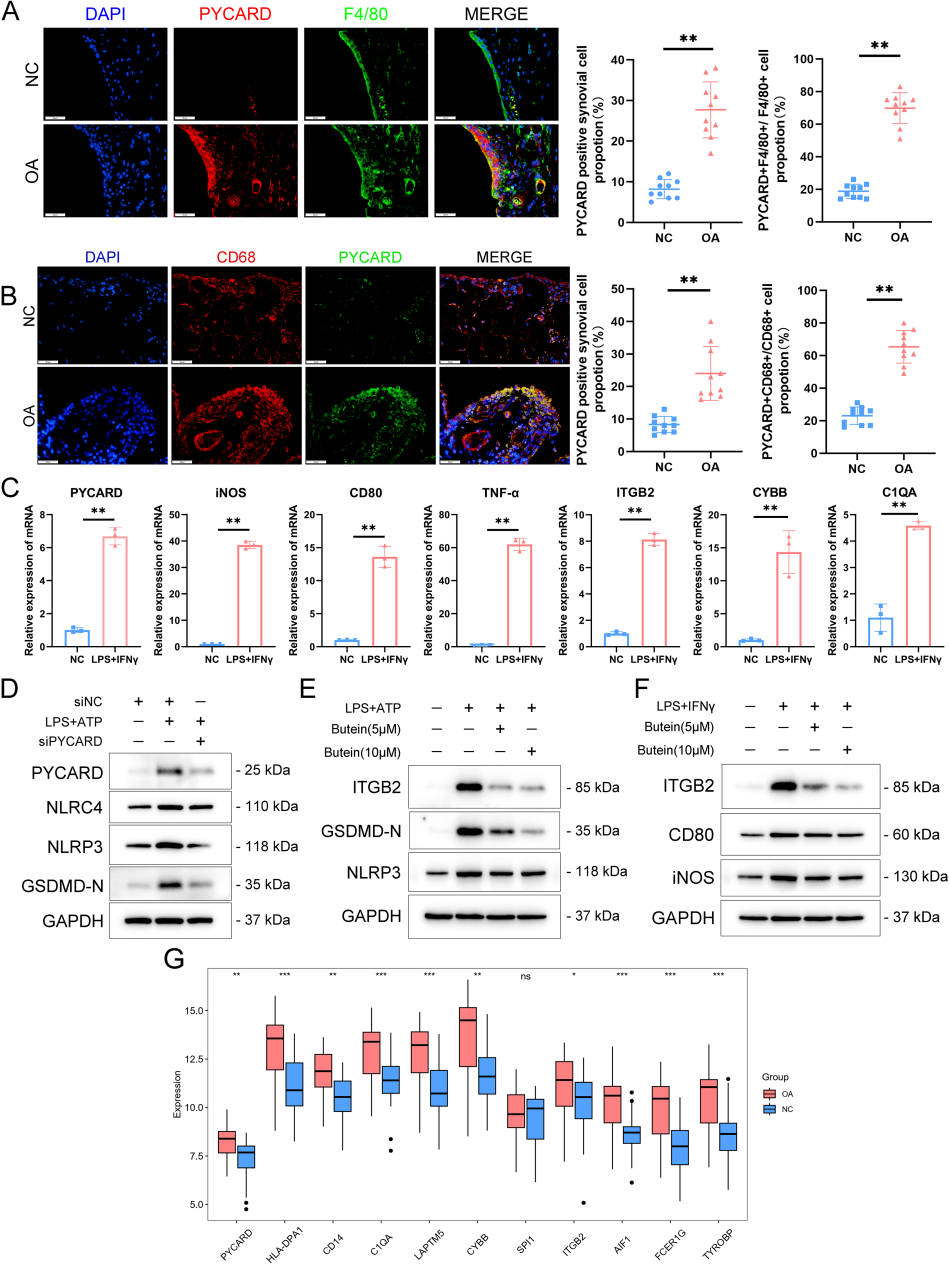
PYCARD was upregulated in the OA synovium. **(A)** Immunofluorescence staining showing the expression of PYCARD (red), F4/80 (green), and DAPI (blue) in synovial tissue from normal control (NC) and osteoarthritis (OA) samples. The proportion of PYCARD -positive synovial cells (left) and PYCARD -F4/80 positive cells (right) are quantified. n = 10 per group. Scale bar: 50 μm. **(B)** Immunofluorescence staining showing the expression of PYCARD (red), CD68 (green), and DAPI (blue) in synovial tissue from NC and OA samples at the 8-week endpoint post-destabilization of the medial meniscus (DMM). The proportion of PYCARD -positive synovial cells (left) and PYCARD -CD68 positive cells (right) are quantified. n = 10 per group. Scale bar: 50 μm. **(C)** mRNA expression levels of M1 polarization markers and PYCARD-related key gene clusters in NC and LPS+IFNγ-stimulated cells. **(D)** Western blot analysis of PYCARD, NLRC4, NLRP3, and GSDMD-N in macrophages treated with siNC, LPS+ATP, and siPYCARD. **(E)** Western blot analysis of ITGB2, GSDMD-N, and NLRP3 in macrophages treated with LPS+ATP, Butein(5μM),and Butein(10μM). **(F)** Western blot analysis of ITGB2, CD80, and iNOS in macrophages treated with LPS and IFN-γ, Butein(5μM),and Butein(10μM). **(G)** Boxplot showing the expression levels of PYCARD-related key gene clusters in the GSE89408 dataset. Data are presented as mean ± SD. An unpaired Student’s t-test was used to compare two groups of data. *P < 0.05, **P < 0.01, *** p < 0.001, ns, not significant.

To verify PYCARD expression levels and its localization in the synovium, a destabilization of the medial meniscus model was developed in 10 mice, and PYCARD expression was analyzed in the synovium 8 weeks following surgery. PYCARD expression was significantly elevated in the synovium of OA mice and showed strong colocalization with the macrophage marker F4/80 (**Figure 8A**). Samples of synovial OA from clinical cases (n = 10) and control synovial samples from patients having arthroscopy due to trauma or joint issues (n = 10) were gathered. There were no notable differences in age, sex, or BMI between the two groups (**Table 1**). As anticipated, elevated PYCARD expression was observed in OA samples, with significant colocalization with CD68, a macrophage marker (**Figure 8B**). We further stimulated THP-1 cells with LPS and IFN-γ to model M1 macrophages and performed qPCR validation of PYCARD-related core genes. Compared to the NC group, macrophages stimulated with LPS and IFN-γ exhibited increased expression of iNOS, CD80, and TNFα, confirming the successful induction of M1 polarization. Furthermore, M1-polarized macrophages exhibited an increase in PYCARD, TYROBP, FCER1G, AIF1, ITGB2, SPI1, CYBB, LAPTM5, C1QA, CD14, HLA-DPA1, and AIF1 (**Figure 8C**, **Supplementary Figure S1E**). We found that PYCARD knockdown attenuated the expression of macrophage inflammasome and pyroptosis indicators, including NLRP3, NLRC4, and GSDMD-N (**Figure 8D**). Moreover, we verified that Butein alleviated the LPS+ATP-induced macrophage pyroptosis process by reducing NLRP3 and GSDMD-N expression and inhibiting the expression of the PYCARD-related hub gene ITGB2 predicted by the DGIdb database (**Figure 8E**). Additionally, we found that Butein alleviated LPS and IFN-γ-stimulated expression of iNOS, CD80, and inhibited ITGB2 expression (**Figure 8F**). Furthermore, we examined gene expression levels in OA patient samples using the GSE89408 dataset. With the exception of SPI1, most dataset results aligned with our experimental findings (**Figure 8G**).

## 4 Discussion

Pyroptosis is an inflammatory cell death marked by unique morphological changes (39). Alterations in pyroptosis-related molecules and inflammatory factors have been observed in both OA animal models and the joints of OA patients (12). The involvement of pyroptosis-related genes in OA, particularly in synovitis, is not yet fully elucidated.

Initially, we examined the expression of genes associated with pyroptosis in OA. In the GSE89408 dataset, 20 out of 57 pyroptosis-related genes, including CASP4, CASP1, NLRC4, CASP8, IL1B, IL18, CASP5, CASP3, DHX8, TNF, PYCARD, NOD2, IL6, CASP6, IRF2, IL1A, CYCS, CHMP2B, CHMP3, and CHMP2A, were found to be upregulated in OA tissues compared to normal ones. A significant positive correlation between the expression levels of CASP3 and CASP4 was found in the analysis of 20 pyroptosis-related DEGs across the entire dataset and OA samples from GSE89408. Caspases are evolutionarily conserved cysteine-dependent endoproteases that specifically cleave substrates at aspartic acid residues (40). Caspases are categorized into apoptotic and inflammatory types based on their structural and functional characteristics. Apoptotic caspases are categorized into initiator caspases, such as caspase-8, -9, and -10, which trigger apoptosis, and executioner caspases, including caspase-3, -6, and -7, responsible for executing the apoptotic process. Inflammatory caspases, including caspase-1, -4, -5, and -11, are involved in pyroptosis and the secretion of inflammatory cytokines. These caspases possess a caspase activation and recruitment domain at their N-terminus, connected to a protease domain via a CARD linker (41). The protease domain comprises large and small catalytic subunits, which are divided by interdomain linkers. Caspase-1, the primary effector protease in the canonical pathway, aggregates on the inflammasome platform by interacting with adaptor proteins like ASC or NLRC4. Human caspases-4 and -5, as well as their murine counterpart caspase-11, necessitate dimerization for protease activation. Caspase-4, -5, and -11 CARDs directly interact with LPS or endogenous oxidized phospholipids to form noncanonical inflammasomes, which are lipid-protein assemblies, unlike caspase-1 (42, 43). Inflammatory caspases, despite their limited ability to cleave pro-IL-1β or pro-IL-18, trigger pyroptosis by cleaving Gasdermin-D proteins (44). The IL-1 family is strongly associated with destructive inflammation and pyroptosis, with significant focus on IL-1β’s central role in mediating inflammatory diseases. Pro-IL-1β remains in the cytoplasm in an inactive state until cleaved into its active form by NLRP3 and caspase-1 activation (45). IRF-2 is crucial for activating GSDMD transcription, which is necessary for triggering pyroptotic cell death. Our study identified an upregulation of caspase family members (CASP4, CASP1, CASP8, CASP5, CASP3, and CASP6), inflammatory cytokines (TNF, IL6, IL1B, and IL1A), and associated factors in the synovium of OA patients. These findings indicate the occurrence of pyroptosis in the synovium during OA.

Considering the significant role of pyroptosis in OA development, we developed a predictive model using 20 differentially expressed genes related to pyroptosis. The final model incorporated six genes (IL1B, DHX8, PYCARD, IL6, CYCS, and CHMP2B). ROC curve analysis revealed strong diagnostic value for these genes, with IL-1B, PYCARD, IL-6, CYCS, and CHMP2B showing an AUC > 0.7. Hazard ratios demonstrated a significant link between PYCARD and elevated OA risk. PYCARD, or apoptosis-associated speck-like protein with a caspase recruitment domain (ASC), was first discovered in human promyelocytic leukemia cells undergoing apoptosis after treatment with etoposide and all-trans retinoic acid (46). PYCARDs are involved in p53-Bax-dependent apoptosis through the mediation of caspase-2, caspase-3, and caspase-9 (47). Zhang et al. verified that inhibition of macrophage PYCARD oligomerization blocked NLRP3 inflammasome activation and alleviated OA (48). Another study found that Degrasyn selectively impedes the form of ASC oligomer and speckle to effectively suppress the NLRP3 inflammasome, alleviate the GSDMD-mediated pyroptosis of macrophages and the release of IL-1β, caspase-1, and LDH in OA (49). Moreover, Chen et al. revealed that AMPK activation in chondrocytes alleviates OA chondrocyte pyroptosis by inhibiting NLRP3 inflammasomes ASC, Caspase-1, IL-1β, NLRP3, and cleaved Caspase-1 (50). These findings highlight the essential function of PYCARD in OA-related pyroptosis.

To determine the localization of the six pyroptosis-related genes, we performed joint analysis of the scRNA-seq data. Our results revealed that CYCS and CHMP2B were predominantly expressed in fibroblasts, whereas CHMP2B, CYCS, IL1B, and PYCARD were highly expressed in macrophages. Notably, among all the genes, PYCARD presented the highest expression in macrophages. Pyroptosis is typically observed in myeloid lineage professional phagocytes, including macrophages, dendritic cells, and neutrophils. This phenomenon has also been noted in various cell types such as keratinocytes, epithelial cells, endothelial cells, and neurons (51). When faced with intense stimuli, macrophages can either directly undergo pyroptosis or become proinflammatory phenotypes that secrete cytokines, leading to pyroptosis in adjacent cells. In macrophages, the NLRP3 inflammasome functions as a stress sensor, identifying cellular and tissue stress and converting it into an inflammatory response. Although these responses aid in defense and stress adaptation, they may become maladaptive under chronic stress, causing pathological inflammatory changes in bones and joints (52). In the context of OA, research has suggested that macrophages in the synovium deposit crystals. The NLRP3 inflammasome is activated by these crystals and ATP released from dead cells, leading to the production of inflammatory cytokines IL-1β and IL-18 (53). The findings indicate that PYCARD is involved in macrophage pyroptosis during synovial inflammation in OA.

Cells were categorized into high- and low-PYCARD expression groups based on their PYCARD expression levels, followed by differential analysis. We identified 128 DEGs and performed GO and KEGG analyses on the upregulated and downregulated genes. Numerous GO terms and KEGG pathways associated with MHC class II and antigen processing and presentation suggest the involvement of immune response pathways in regulating pyroptosis and PYCARD expression.

We developed a PPI network for the 128 DEGs and utilized cytoHubba to identify 10 key genes: TYROBP, FCER1G, AIF1, ITGB2, SPI1, CYBB, LAPTM5, C1QA, CD14, and HLA-DPA1. We predicted drug-gene interactions to identify potential therapeutic candidates targeting these key genes. The membrane-encoded adaptor protein TYROBP plays a vital role in the transduction of immune signals (54). The regulation of the immune system, especially in the monocyte-macrophage system, heavily relies on TYROBP, which influences the proliferation, survival, differentiation, and polarization of immune cells. Through the activation of TYROBP-related signaling pathways, Triggering Receptor Expressed on Myeloid cells-1 facilitates the production of proinflammatory cytokines and chemokines in hypoxic mature dendritic cells (46). Furthermore, according to previous reports, TYROBP may mediate pathological changes in OA through proinflammatory mechanisms (55). Following mechanical injury in mice, FCER1G, a vital part of the high-affinity IgE receptor, may activate mast cells via IgE, causing synovitis and cartilage damage in osteoarthritis (56). Another study revealed that IgE contributes to atherosclerotic foam cell formation by modulating the polarization state of macrophages (57). ITGB2, a subunit of integrin, is a surface receptor expressed specifically on leukocytes that forms a heterodimer (58). ITGB2 expression is linked to diverse blood cell types and may play a crucial role in regulating immune cell infiltration and inflammation (59). Previous studies have also suggested that ITGB2 is associated with the M1 polarization of macrophages (60). Recent studies have identified ITGB2 as a contributor to OA progression, noting its increased expression in OA tissues (61). SPI1, part of the E26 transformation-specific transcription factor family, plays a vital role in the differentiation and function of various myeloid cells (62). A prior study indicated that SPI1 could serve as a therapeutic target in rheumatoid arthritis by directly inhibiting Feline Mcdonough sarcoma-like tyrosine kinase 3 (63). Recent studies indicate that SPI1-induced ADAMTS5 expression is crucial in OA, and overexpressing Dnmt1 to suppress ADAMTS5 significantly alleviates cartilage damage in OA mice (64). CYBB is the final element of a respiratory chain that facilitates the transfer of electrons from cytoplasmic NADPH across the plasma membrane to extracellular molecular oxygen. It encodes the β-chain of flavocytochrome b245 (gp91phox or NOX2), a crucial component of the NADPH oxidase complex in phagocytes such as granulocytes, monocytes, and macrophages (65). Recent research indicates that nimesulide could potentially treat OA by reducing chondrocyte necroptosis via CYBB gene expression downregulation (66). C1q is a key element of the classical complement activation pathway, linking innate immunity with acquired immunity through IgG or IgM (67). Studies have shown that C1q is associated with the number of polarized M1 macrophages (68). Additionally, recent research suggests that C1QA can be utilized for diagnosing OA and aiding in clinical decision-making (69). The HLA-DPA1 gene, part of the major histocompatibility complex class II, potentially regulates OA by presenting antigens via MHC II molecules. Type II collagen-specific T regulatory cells in the OA synovium are activated when Col II is presented on MHC II by antigen-presenting cells, including macrophages and dendritic cells (70). Studies indicate that HLA-DPA1, linked to the OA immune microenvironment, demonstrates strong diagnostic capabilities and is a potential biomarker (56). Although there are no specific reports on LAPTM5 and AIF1 in the context of OA, the literature suggests that both are highly associated with inflammation (71). By analyzing drug-gene interactions for 10 key genes via the DGIdb database, we identified 57 potential drugs or compounds for OA treatment. A review of the pharmacological effects of these compounds revealed that they primarily consist of immunomodulators, including Therapeutic Immune Globulin, Erlizumab, and Visilizumab, as well as flavonoid anti-inflammatory agents such as Chrysin, Apigenin, and Luteolin. In the field of immunomodulation, Yacov’s team demonstrated the efficacy of VB-201 in alleviating glomerular damage, glomerulosclerosis, and fibrosis in rats through the inhibition of monocyte migration (72). Ahmad et al. found that Chrysin reduced the production of macrophage inflammatory mediators IL-6 and TNF-α by targeting DPP-8/9 (73). Additionally, Chen’s team demonstrated that Chrysin-loaded macrophage-targeting nano- and nanocellular carcinoma (MCAC) carriers inhibited M1 polarization and alleviated OA (74). Lauritzen’s team reported that Apigenin inhibited the production of NLRP3 inflammasomes and the inflammatory cytokine IL-1β during macrophage pyroptosis through the inhibition of CD38 (75). Several studies have suggested that Luteolin promotes M2 macrophage polarization and reduces the release of pro-inflammatory factors by inhibiting macrophage PI3K-AKT/NF-κB/MAPK and JAK2/STAT3 signaling pathways (76–79). Notably, Makino et al. directly verified that Luteolin, Chrysin, and Apigenin inhibited THP-1 cell differentiation by suppressing the expression of CYBB, a key target screened by the DGIdb database (76). And Kojima et al. demonstrated that Butein reduces intercellular adhesion molecule-1 (ICAM-1) expression by inhibiting NF-κB and AP-1 activation, with ITGB2 acting as the binding ligand for ICAM-1 (80). Similarly, a study indicated that Butein blocks NLRP3 inflammasome activation in mouse macrophages by inhibiting ASC oligomerization, reactive oxygen species production, and upregulation of the antioxidant pathway nuclear factor erythroid 2-related factor 2 (Nrf2) (81). In addition, we verified that Butein alleviated the LPS+ATP-induced macrophage pyroptosis process NLRP3 and GSDMD-N expression and inhibited the expression of PYCARD-related hub gene ITGB2 predicted by DGIdb database. In addition, we also found that Butein alleviated LPS and IFN-γ stimulated expression of macrophage M1 polarization indicators (iNOS, CD86) and likewise inhibited ITGB2 expression. This suggests that Butein may regulate macrophage inflammatory polarization and pyroptosis by modulating ITGB2, however the exact mechanism of regulation requires further investigation. These findings highlight the potential of these compounds as therapeutic agents for the treatment of OA.

We performed clustering of the 113 synovial samples based on PYCARD and its 10 associated key DEGs. The samples were divided into two separate clusters, C1 (N = 49) and C2 (N = 64), based on the highest consensus values. The C2 cluster demonstrated a stronger association with OA characteristics in comparison to the C1 cluster. Furthermore, an analysis of intercluster expression of PYCARD and its core DEGs revealed significantly elevated expression levels in the C2 cluster. The C2 cluster demonstrated increased immune cell infiltration, particularly with a higher proportion of macrophages (M1 and M2 subtypes), while fibroblast numbers remained comparable between the two clusters. These findings suggest that synovial macrophage pyroptosis in OA should be a focal point of further research, given its potential role in disease pathogenesis and as a therapeutic target.

We assessed PYCARD expression under OA conditions to validate our findings. Initially, we induced medial meniscus destabilization in a cohort of 10 mice for experimental analysis. PYCARD exhibited high expression levels in the synovium of OA mice and demonstrated significant colocalization with the macrophage marker F4/80. Furthermore, OA synovial macrophages in human synovial samples exhibited a marked increase in PYCARD expression, which showed strong colocalization with the macrophage marker CD68. Previous studies have confirmed that M1 inflammatory polarization of synovial tissue macrophages plays a key role in accelerating the pathological progression of OA (53, 82, 83). Targeting M1 inflammatory macrophages may, therefore, serve as a therapeutic strategy to alleviate OA (8, 84). The LPS and IFN-γ protocol, commonly used to induce macrophage M1 polarization, is recognized as a model for generating a typical pro-inflammatory phenotype (85–87). Although this cellular model does not fully replicate the complex environment of OA, it partially mimics the pathology of OA-related synovial inflammation. We observed that LPS and IFN-γ induced M1 macrophages exhibited high expression levels of PYCARD, TYROBP, FCER1G, AIF1, ITGB2, SPI1, CYBB, LAPTM5, C1QA, CD14, and HLA-DPA1. Previous studies suggest that PYCARD is significantly associated with macrophage polarization towards the M1 phenotype (88). Our subsequent validation further confirmed that M1-polarized macrophages exhibit high expression levels of PYCARD and its associated core genes. These findings suggest that PYCARD and its core-related genes may serve as key regulatory factors for M1 macrophages, warranting further investigation. These findings suggest that PYCARD may function as both a biomarker and a therapeutic target for OA synovium.

Previous studies have employed bioinformatics to explore the expression patterns of genes associated with pyroptosis in OA, with particular emphasis on cartilage pyroptosis (89). They concluded that PYCARD could serve as a diagnostic marker. However, current research on PYCARD in macrophages remains limited, with only a few studies addressing its role in macrophages within OA synovial inflammation (49, 90). We verified that knockdown of PYCARD in macrophages alleviated LPS- and ATP-induced expression of GSDMD-N, NLRC4, and NLRP3, which partially validated that PYCARD regulates macrophage pyroptosis through the canonical pyroptosis pathway. Our study offers unique advantages and novelty compared to previous research. We assembled a comprehensive collection of public RNA-seq datasets from synovial samples, including 55 from healthy individuals and 57 from those with OA, to bolster the reliability of our bioinformatics analysis. We developed an OA prediction model using LASSO regression and hazard ratios (HRs), based on the expression of pyroptosis-related genes. Furthermore, our study involved a combined and pioneering analysis of bulk RNA-seq and scRNA-seq data, enabling the discovery of pyroptosis-related gene expression patterns in each synovial cell cluster in OA for the first time. Additionally, we identified PYCARD as specifically expressed in OA synovial macrophages. Moreover, based on genes related to PYCARD, we screened potential therapeutic drugs from the DGIdb database, providing support for OA treatment. Experiments were performed to verify the clinical relationship between PYCARD levels and OA, and to investigate its expression and related genes in synovial macrophage polarization.

Nevertheless, several limitations are present in our study. First, the deconvolution of normal synovial samples could be biased due to missing scRNA-seq data for normal synovium. Secondly, online tools were used to predict drugs for PYCARD, and experimental validation is needed for the interactions between these drugs and PYCARD or its associated genes. Third, our study currently lacks PYCARD knockout transgenic mice, which we plan to incorporate in future research to strengthen our conclusions.

## 5 Conclusions

We conducted a comprehensive bioinformatics study to pinpoint crucial genes associated with pyroptosis in individuals with OA. Our results also identified 57 potential therapeutic agents or compounds for OA, which were connected to significant genes related to PYCARD. Additional research is required to confirm the efficacy of these agents in OA treatment.

## Data Availability

All data used in this study were obtained from the NCBI Gene Expression Omnibus (GEO) database. The specific dataset information is as follows: Synovial bulk RNA-seq datasets: GSE55235 (10 healthy and 10 OA synovium samples); GSE55457 (10 healthy and 10 OA synovium samples); GSE82107 (7 healthy and 10 OA synovium samples); GSE55584 (6 OA synovium samples); GSE89408 (28 healthy and 22 OA synovium samples). Synovial single-cell RNA-seq dataset: GSE152805 (3 OA synovium samples). All datasets can be accessed at https://www.ncbi.nlm.nih.gov/geo/.
