# Supplementary material for: Comprehensive multiomics analysis identifies PYCARD as a key pyroptosis-related gene in osteoarthritis synovial macrophages
Source: Front Immunol. 2025 Mar 24;16:1558139. doi: 10.3389/fimmu.2025.1558139 (PMC11973068; doi:10.3389/fimmu.2025.1558139)
Supplement: Supplementary file 1 [file DataSheet1.docx]

Table S1 Pyroptosis-related genes

| **Gene** | **Full name** |
| --- | --- |
| BAK1 | Bcl-2 homologous antagonist/killer 1 |
| BAX | Bcl-2-associated X protein |
| CHMP2A | Charged multivesicular body protein 2A |
| CHMP2B | Charged multivesicular body protein 2B |
| CHMP3 | Charged multivesicular body protein 3 |
| CHMP4A | Charged multivesicular body protein 4A |
| CHMP4B | Charged multivesicular body protein 4B |
| CHMP4C | Charged multivesicular body protein 4C |
| CHMP6 | Charged multivesicular body protein 6 |
| CHMP7 | Charged multivesicular body protein 7 |
| CYCS | Cytochrome c, somatic |
| HMGB1 | High mobility group box 1 |
| IL1A | Interleukin-1 alpha |
| IRF1 | Interferon regulatory factor 1 |
| IRF2 | Interferon regulatory factor 2 |
| TP53 | Tumor protein p53 |
| TP63 | Tumor protein p63 |
| CASP6 | Caspase 6 |
| CASP9 | Caspase 9 |
| GPX4 | Glutathione peroxidase 4 |
| IL6 | Interleukin-6 |
| NLRP2 | NLR family pyrin domain containing 2 |
| NLRP3 | NLR family pyrin domain containing 3 |
| NLRP6 | NLR family pyrin domain containing 6 |
| NLRP7 | NLR family pyrin domain containing 7 |
| NOD1 | Nucleotide-binding oligomerization domain-containing protein 1 |
| NOD2 | Nucleotide-binding oligomerization domain-containing protein 2 |
| PJVK | Pejvakin |
| PLCG1 | Phospholipase C gamma 1 |
| PRKACA | Protein kinase cAMP-activated catalytic subunit alpha |
| PYCARD | PYD and CARD domain containing |
| SCAF11 | Scaffold attachment factor B |
| TIRAP | TIR domain-containing adapter protein |
| TNF | Tumor necrosis factor |
| APIP | Apoptosis-inducing protein |
| DHX9 | DEAH-box helicase 9 |
| GZMA | Granzyme A |
| NAIP | NLR family apoptosis inhibitory protein |
| NLRP9 | NLR family pyrin domain containing 9 |
| ZBP1 | Z-DNA binding protein 1 |
| CASP3 | Caspase 3 |
| CASP5 | Caspase 5 |
| ELANE | Neutrophil elastase |
| IL18 | Interleukin-18 |
| IL1B | Interleukin-1 beta |

Table S2. List of 128 differentially expressed genes.

|  | **Gene** | **p_val** | **avg_log2FC** | **pct.1** | **pct.2** | **p_val_adj** | **symbol** | **threshold** | **Difference** |
| --- | --- | --- | --- | --- | --- | --- | --- | --- | --- |
| 1 | PYCARD | 1.73E-210 | 1.677154792 | 1 | 0.276 | ####### | PYCARD | up | 0.724 |
| 2 | C1QA | 6.40E-33 | 0.773465644 | 0.842 | 0.578 | 1.22E-28 | C1QA | up | 0.264 |
| 3 | C1QC | 2.25E-32 | 0.797050329 | 0.807 | 0.534 | 4.29E-28 | C1QC | up | 0.273 |
| 4 | AIF1 | 3.06E-31 | 0.743706099 | 0.807 | 0.533 | 5.84E-27 | AIF1 | up | 0.274 |
| 5 | FOLR2 | 5.59E-31 | 0.798734107 | 0.765 | 0.486 | 1.07E-26 | FOLR2 | up | 0.279 |
| 6 | FCER1G | 2.53E-30 | 0.724286433 | 0.82 | 0.566 | 4.82E-26 | FCER1G | up | 0.254 |
| 7 | TGFBI | 3.04E-29 | 0.668748187 | 0.87 | 0.679 | 5.79E-25 | TGFBI | up | 0.191 |
| 8 | TYROBP | 5.13E-29 | 0.678088756 | 0.833 | 0.584 | 9.78E-25 | TYROBP | up | 0.249 |
| 9 | CRTAC1 | 9.03E-29 | -1.33716508 | 0.714 | 0.839 | 1.72E-24 | CRTAC1 | down | -0.125 |
| 10 | CST3 | 2.79E-28 | 0.54418554 | 1 | 0.999 | 5.31E-24 | CST3 | up | 0.001 |
| 11 | RNASE1 | 6.51E-28 | 0.745831518 | 0.834 | 0.609 | 1.24E-23 | RNASE1 | up | 0.225 |
| 12 | FN1 | 7.91E-28 | -1.028674431 | 0.899 | 0.94 | 1.51E-23 | FN1 | down | -0.041 |
| 13 | CYBA | 8.44E-28 | 0.594823003 | 0.953 | 0.88 | 1.61E-23 | CYBA | up | 0.073 |
| 14 | ERRFI1 | 3.31E-27 | -1.176497277 | 0.445 | 0.654 | 6.31E-23 | ERRFI1 | down | -0.209 |
| 15 | MS4A6A | 4.20E-27 | 0.628861407 | 0.779 | 0.49 | 8.00E-23 | MS4A6A | up | 0.289 |
| 16 | TIMP3 | 4.27E-27 | -1.266100858 | 0.469 | 0.668 | 8.13E-23 | TIMP3 | down | -0.199 |
| 17 | INHBA | 6.89E-27 | -1.219243399 | 0.308 | 0.566 | 1.31E-22 | INHBA | down | -0.258 |
| 18 | ALOX5AP | 1.08E-26 | 0.636391759 | 0.717 | 0.442 | 2.06E-22 | ALOX5AP | up | 0.275 |
| 19 | PROCR | 1.55E-26 | -0.723598353 | 0.231 | 0.484 | 2.96E-22 | PROCR | down | -0.253 |
| 20 | SPARCL1 | 3.70E-26 | -1.232525571 | 0.423 | 0.632 | 7.04E-22 | SPARCL1 | down | -0.209 |
| 21 | C2orf40 | 3.90E-26 | -1.20496223 | 0.294 | 0.53 | 7.44E-22 | C2orf40 | down | -0.236 |
| 22 | VAMP8 | 1.57E-25 | 0.621830946 | 0.774 | 0.543 | 2.98E-21 | VAMP8 | up | 0.231 |
| 23 | CYBB | 2.42E-25 | 0.631351225 | 0.72 | 0.447 | 4.61E-21 | CYBB | up | 0.273 |
| 24 | CLU | 3.16E-25 | -1.163881866 | 0.847 | 0.89 | 6.02E-21 | CLU | down | -0.043 |
| 25 | C1QB | 3.40E-25 | 0.648412501 | 0.806 | 0.568 | 6.47E-21 | C1QB | up | 0.238 |
| 26 | TNFAIP6 | 3.63E-25 | -1.161071127 | 0.417 | 0.648 | 6.92E-21 | TNFAIP6 | down | -0.231 |
| 27 | PLA2G2A | 5.39E-25 | -1.038966262 | 0.7 | 0.842 | 1.03E-20 | PLA2G2A | down | -0.142 |
| 28 | VSIG4 | 1.30E-24 | 0.64153532 | 0.716 | 0.457 | 2.48E-20 | VSIG4 | up | 0.259 |
| 29 | AK1 | 2.09E-24 | -0.925068665 | 0.428 | 0.626 | 3.97E-20 | AK1 | down | -0.198 |
| 30 | CTSZ | 2.71E-24 | 0.618523985 | 0.853 | 0.648 | 5.17E-20 | CTSZ | up | 0.205 |
| 31 | DEFB1 | 4.05E-24 | -1.047087309 | 0.254 | 0.485 | 7.71E-20 | DEFB1 | down | -0.231 |
| 32 | CD74 | 4.71E-24 | 0.660797309 | 0.889 | 0.714 | 8.98E-20 | CD74 | up | 0.175 |
| 33 | MARCO | 5.18E-24 | 0.632238427 | 0.766 | 0.491 | 9.88E-20 | MARCO | up | 0.275 |
| 34 | MGP | 6.07E-24 | -0.968483122 | 0.728 | 0.838 | 1.16E-19 | MGP | down | -0.11 |
| 35 | MT2A | 7.52E-24 | -0.998375719 | 0.842 | 0.897 | 1.43E-19 | MT2A | down | -0.055 |
| 36 | PRG4 | 9.24E-24 | -1.222113304 | 0.875 | 0.938 | 1.76E-19 | PRG4 | down | -0.063 |
| 37 | LAPTM5 | 1.01E-23 | 0.565403414 | 0.82 | 0.563 | 1.92E-19 | LAPTM5 | up | 0.257 |
| 38 | VASN | 1.41E-23 | -0.740600728 | 0.28 | 0.507 | 2.69E-19 | VASN | down | -0.227 |
| 39 | LUM | 2.07E-23 | -1.02081845 | 0.739 | 0.854 | 3.94E-19 | LUM | down | -0.115 |
| 40 | STAB1 | 3.88E-23 | 0.614758463 | 0.643 | 0.391 | 7.39E-19 | STAB1 | up | 0.252 |
| 41 | ANGPTL2 | 3.97E-23 | -0.846079982 | 0.355 | 0.581 | 7.57E-19 | ANGPTL2 | down | -0.226 |
| 42 | CD14 | 3.98E-23 | 0.644073161 | 0.844 | 0.679 | 7.58E-19 | CD14 | up | 0.165 |
| 43 | NCF1 | 4.41E-23 | 0.72143646 | 0.611 | 0.36 | 8.41E-19 | NCF1 | up | 0.251 |
| 44 | MT1M | 8.10E-23 | -0.851082795 | 0.27 | 0.511 | 1.54E-18 | MT1M | down | -0.241 |
| 45 | HTRA1 | 8.65E-23 | -0.975396891 | 0.731 | 0.813 | 1.65E-18 | HTRA1 | down | -0.082 |
| 46 | CAV1 | 9.02E-23 | -0.717145735 | 0.311 | 0.566 | 1.72E-18 | CAV1 | down | -0.255 |
| 47 | PCOLCE2 | 2.02E-22 | -0.769689492 | 0.321 | 0.547 | 3.86E-18 | PCOLCE2 | down | -0.226 |
| 48 | HAS1 | 2.35E-22 | -1.06682013 | 0.338 | 0.541 | 4.48E-18 | HAS1 | down | -0.203 |
| 49 | EMB | 3.13E-22 | 0.552318889 | 0.596 | 0.349 | 5.97E-18 | EMB | up | 0.247 |
| 50 | FCGR2A | 3.41E-22 | 0.539301652 | 0.687 | 0.452 | 6.50E-18 | FCGR2A | up | 0.235 |
| 51 | GPRC5A | 4.29E-22 | -0.638971502 | 0.327 | 0.568 | 8.16E-18 | GPRC5A | down | -0.241 |
| 52 | SPI1 | 5.25E-22 | 0.626398326 | 0.55 | 0.323 | 9.99E-18 | SPI1 | up | 0.227 |
| 53 | UAP1 | 5.74E-22 | -0.749802571 | 0.444 | 0.644 | 1.09E-17 | UAP1 | down | -0.2 |
| 54 | ITGB2 | 1.62E-21 | 0.516912767 | 0.607 | 0.367 | 3.08E-17 | ITGB2 | up | 0.24 |
| 55 | NNMT | 4.50E-21 | -0.840351839 | 0.45 | 0.647 | 8.58E-17 | NNMT | down | -0.197 |
| 56 | SEPP1 | 4.51E-21 | 0.755654219 | 0.908 | 0.834 | 8.59E-17 | SEPP1 | up | 0.074 |
| 57 | SLC39A14 | 5.27E-21 | -0.559950652 | 0.265 | 0.5 | 1.00E-16 | SLC39A14 | down | -0.235 |
| 58 | CTSS | 8.38E-21 | 0.543942995 | 0.818 | 0.64 | 1.60E-16 | CTSS | up | 0.178 |
| 59 | CTSD | 1.11E-20 | 0.543007135 | 0.942 | 0.883 | 2.11E-16 | CTSD | up | 0.059 |
| 60 | LYVE1 | 2.02E-20 | 0.758037118 | 0.588 | 0.36 | 3.85E-16 | LYVE1 | up | 0.228 |
| 61 | LGMN | 4.42E-20 | 0.59567949 | 0.791 | 0.641 | 8.42E-16 | LGMN | up | 0.15 |
| 62 | HLA-DRB5 | 4.89E-20 | 0.518030891 | 0.761 | 0.51 | 9.31E-16 | HLA-DRB5 | up | 0.251 |
| 63 | F13A1 | 5.51E-20 | 0.631314751 | 0.555 | 0.332 | 1.05E-15 | F13A1 | up | 0.223 |
| 64 | C11orf96 | 7.80E-20 | -0.59258941 | 0.227 | 0.446 | 1.49E-15 | C11orf96 | down | -0.219 |
| 65 | UGDH | 8.39E-20 | -0.680259474 | 0.359 | 0.557 | 1.60E-15 | UGDH | down | -0.198 |
| 66 | TNXB | 1.11E-19 | -0.574652494 | 0.327 | 0.559 | 2.11E-15 | TNXB | down | -0.232 |
| 67 | LDHA | 1.15E-19 | -0.629063279 | 0.842 | 0.896 | 2.19E-15 | LDHA | down | -0.054 |
| 68 | SLC40A1 | 1.51E-19 | 0.669655329 | 0.504 | 0.279 | 2.87E-15 | SLC40A1 | up | 0.225 |
| 69 | FCGR2B | 1.76E-19 | 0.559370044 | 0.58 | 0.362 | 3.35E-15 | FCGR2B | up | 0.218 |
| 70 | HLA-DMA | 1.97E-19 | 0.537580147 | 0.731 | 0.498 | 3.75E-15 | HLA-DMA | up | 0.233 |
| 71 | HBEGF | 4.25E-19 | -0.706700352 | 0.436 | 0.634 | 8.10E-15 | HBEGF | down | -0.198 |
| 72 | HLA-DRB1 | 4.45E-19 | 0.506784604 | 0.812 | 0.579 | 8.47E-15 | HLA-DRB1 | up | 0.233 |
| 73 | MFAP4 | 5.27E-19 | -0.739864697 | 0.239 | 0.446 | 1.00E-14 | MFAP4 | down | -0.207 |
| 74 | GMFG | 5.46E-19 | 0.551157428 | 0.646 | 0.433 | 1.04E-14 | GMFG | up | 0.213 |
| 75 | CPVL | 5.53E-19 | 0.519487151 | 0.57 | 0.342 | 1.05E-14 | CPVL | up | 0.228 |
| 76 | GFPT2 | 8.19E-19 | -0.684191391 | 0.325 | 0.527 | 1.56E-14 | GFPT2 | down | -0.202 |
| 77 | MT1E | 1.29E-18 | -0.720661133 | 0.389 | 0.592 | 2.46E-14 | MT1E | down | -0.203 |
| 78 | SBDS | 1.30E-18 | -0.536749684 | 0.559 | 0.719 | 2.47E-14 | SBDS | down | -0.16 |
| 79 | YBX1 | 1.65E-18 | 0.596238695 | 0.919 | 0.918 | 3.15E-14 | YBX1 | up | 0.001 |
| 80 | SMIM14 | 2.21E-18 | -0.653375081 | 0.417 | 0.599 | 4.21E-14 | SMIM14 | down | -0.182 |
| 81 | HLA-DMB | 2.58E-18 | 0.500913873 | 0.592 | 0.369 | 4.92E-14 | HLA-DMB | up | 0.223 |
| 82 | CYR61 | 2.73E-18 | -0.609935975 | 0.275 | 0.478 | 5.20E-14 | CYR61 | down | -0.203 |
| 83 | CALD1 | 3.20E-18 | -0.564774231 | 0.34 | 0.572 | 6.11E-14 | CALD1 | down | -0.232 |
| 84 | THBS4 | 3.78E-18 | -0.528719684 | 0.272 | 0.488 | 7.20E-14 | THBS4 | down | -0.216 |
| 85 | GEM | 3.82E-18 | -0.641614246 | 0.483 | 0.673 | 7.29E-14 | GEM | down | -0.19 |
| 86 | CCDC80 | 4.89E-18 | -0.717939655 | 0.392 | 0.588 | 9.31E-14 | CCDC80 | down | -0.196 |
| 87 | NDUFA4L2 | 1.16E-17 | -0.902519457 | 0.343 | 0.538 | 2.22E-13 | NDUFA4L2 | down | -0.195 |
| 88 | IGFBP5 | 1.21E-17 | -0.772404995 | 0.28 | 0.491 | 2.31E-13 | IGFBP5 | down | -0.211 |
| 89 | GPX3 | 1.22E-17 | -0.623269789 | 0.556 | 0.706 | 2.32E-13 | GPX3 | down | -0.15 |
| 90 | BLVRB | 1.23E-17 | 0.502438203 | 0.782 | 0.626 | 2.34E-13 | BLVRB | up | 0.156 |
| 91 | SDC2 | 2.16E-17 | -0.515870488 | 0.382 | 0.597 | 4.11E-13 | SDC2 | down | -0.215 |
| 92 | DCN | 3.61E-17 | -0.610879478 | 0.733 | 0.835 | 6.87E-13 | DCN | down | -0.102 |
| 93 | ANGPTL4 | 5.00E-17 | -0.803717977 | 0.318 | 0.507 | 9.53E-13 | ANGPTL4 | down | -0.189 |
| 94 | CRYAB | 5.69E-17 | -0.620594171 | 0.283 | 0.478 | 1.08E-12 | CRYAB | down | -0.195 |
| 95 | ABI3BP | 6.10E-17 | -0.530005808 | 0.288 | 0.486 | 1.16E-12 | ABI3BP | down | -0.198 |
| 96 | CD9 | 6.44E-17 | -0.610864171 | 0.882 | 0.929 | 1.23E-12 | CD9 | down | -0.047 |
| 97 | TWISTNB | 6.46E-17 | -0.795861974 | 0.324 | 0.523 | 1.23E-12 | TWISTNB | down | -0.199 |
| 98 | HLA-DPA1 | 6.93E-17 | 0.527224044 | 0.779 | 0.58 | 1.32E-12 | HLA-DPA1 | up | 0.199 |
| 99 | C1R | 8.87E-17 | -0.540004637 | 0.37 | 0.586 | 1.69E-12 | C1R | down | -0.216 |
| 100 | CTGF | 2.78E-16 | -0.65252454 | 0.376 | 0.576 | 5.29E-12 | CTGF | down | -0.2 |
| 101 | C1S | 2.85E-16 | -0.574255664 | 0.414 | 0.611 | 5.43E-12 | C1S | down | -0.197 |
| 102 | MT1X | 8.91E-16 | -0.948024763 | 0.567 | 0.711 | 1.70E-11 | MT1X | down | -0.144 |
| 103 | MT1A | 1.20E-15 | -1.020969522 | 0.258 | 0.439 | 2.29E-11 | MT1A | down | -0.181 |
| 104 | PLAC9 | 1.36E-15 | -0.553830482 | 0.401 | 0.59 | 2.59E-11 | PLAC9 | down | -0.189 |
| 105 | MTRNR2L1 | 2.01E-15 | 0.708112202 | 0.487 | 0.295 | 3.83E-11 | MTRNR2L1 | up | 0.192 |
| 106 | MEG3 | 2.09E-15 | -0.708886978 | 0.246 | 0.43 | 3.98E-11 | MEG3 | down | -0.184 |
| 107 | RGS16 | 2.23E-15 | -0.589734982 | 0.359 | 0.536 | 4.25E-11 | RGS16 | down | -0.177 |
| 108 | UGP2 | 1.17E-14 | -0.574249093 | 0.635 | 0.739 | 2.22E-10 | UGP2 | down | -0.104 |
| 109 | TXNIP | 2.65E-14 | 0.520283598 | 0.621 | 0.462 | 5.05E-10 | TXNIP | up | 0.159 |
| 110 | IGFBP6 | 2.87E-14 | -0.585680523 | 0.439 | 0.627 | 5.47E-10 | IGFBP6 | down | -0.188 |
| 111 | SELM | 4.74E-14 | -0.50687904 | 0.597 | 0.726 | 9.02E-10 | SELM | down | -0.129 |
| 112 | DUSP1 | 6.59E-14 | 0.513873342 | 0.798 | 0.683 | 1.25E-09 | DUSP1 | up | 0.115 |
| 113 | TPPP3 | 8.76E-14 | -0.597382376 | 0.638 | 0.751 | 1.67E-09 | TPPP3 | down | -0.113 |
| 114 | HSPA1A | 9.87E-14 | 0.57398437 | 0.804 | 0.72 | 1.88E-09 | HSPA1A | up | 0.084 |
| 115 | LAPTM4A | 1.62E-13 | -0.523194597 | 0.872 | 0.914 | 3.08E-09 | LAPTM4A | down | -0.042 |
| 116 | CDO1 | 2.47E-13 | -0.659091315 | 0.246 | 0.416 | 4.71E-09 | CDO1 | down | -0.17 |
| 117 | PTPRS | 6.75E-13 | -0.514961343 | 0.161 | 0.318 | 1.29E-08 | PTPRS | down | -0.157 |
| 118 | MYC | 1.87E-12 | -0.591218792 | 0.415 | 0.564 | 3.57E-08 | MYC | down | -0.149 |
| 119 | MIR4435-1HG | 2.08E-12 | -0.517945689 | 0.294 | 0.457 | 3.96E-08 | MIR4435-1HG | down | -0.163 |
| 120 | VCAM1 | 2.56E-11 | -0.542907998 | 0.232 | 0.392 | 4.87E-07 | VCAM1 | down | -0.16 |
| 121 | AXL | 1.07E-10 | -0.503871222 | 0.318 | 0.459 | 2.03E-06 | AXL | down | -0.141 |
| 122 | ID3 | 1.71E-10 | -0.624852248 | 0.436 | 0.569 | 3.25E-06 | ID3 | down | -0.133 |
| 123 | SOCS3 | 5.94E-10 | -0.51132619 | 0.573 | 0.687 | 1.13E-05 | SOCS3 | down | -0.114 |
| 124 | PRELP | 7.68E-10 | -0.698764109 | 0.381 | 0.519 | 1.46E-05 | PRELP | down | -0.138 |
| 125 | ADAMTS1 | 8.41E-10 | -0.531976452 | 0.205 | 0.342 | 1.60E-05 | ADAMTS1 | down | -0.137 |
| 126 | CXCL1 | 1.58E-09 | -0.769039132 | 0.559 | 0.703 | 3.02E-05 | CXCL1 | down | -0.144 |
| 127 | HSPA1B | 6.45E-08 | 0.507444756 | 0.679 | 0.602 | 0.00123 | HSPA1B | up | 0.077 |
| 128 | MTRNR2L2 | 2.09E-07 | 0.551007068 | 0.859 | 0.829 | 0.003978 | hiMTRNR2L2 | up | 0.03 |

Table S3. Top 10 genes with highest Maximal Clique Centrality (MCC) algorithm scores.

| **Rank** | **Name** | **Score** |
| --- | --- | --- |
| 1 | TYROBP | 1300 |
| 2 | FCER1G | 1086 |
| 3 | AIF1 | 1012 |
| 4 | ITGB2 | 870 |
| 5 | SPI1 | 846 |
| 6 | CYBB | 764 |
| 7 | LAPTM5 | 751 |
| 8 | C1QA | 453 |
| 9 | CD14 | 312 |
| 10 | HLA-DPA1 | 276 |

Table S4. 57 potential target drugs/compounds for OA treatment

| **Gene** | **Drug** | **Regulatory approval** | **Interaction score** |
| --- | --- | --- | --- |
| C1QA | THERAPEUTIC IMMUNE GLOBULIN | Not Approved | 52.50767 |
| FCER1G | COMPOUND 66 [PMID: 21802293] | Not Approved | 17.50256 |
| FCER1G | BENZYLPENICILLOYL POLYLYSINE | Approved | 17.50256 |
| CD14 | IC14 | Not Approved | 14.32027 |
| CD14 | NNC-711 | Not Approved | 4.773425 |
| CYBB | COMPOUND 14N [PMID: 25589934] | Not Approved | 4.375639 |
| HLA-DPA1 | PEGINTERFERON ALFA-2B | Approved | 3.281729 |
| ITGB2 | RECOMBINANT C5A | Not Approved | 2.917093 |
| ITGB2 | LYMPHOKINE-ACTIVATED KILLER CELLS | Not Approved | 2.386712 |
| CD14 | (R/S) EF-1500 | Not Approved | 2.386712 |
| CD14 | (R)-EF-1520 | Not Approved | 2.386712 |
| CD14 | SKF89976A | Not Approved | 2.386712 |
| CD14 | (S)-EF-1520 | Not Approved | 2.386712 |
| CYBB | CHRYSIN | Approved | 1.875274 |
| CD14 | TIAGABINE HYDROCHLORIDE | Approved | 1.591142 |
| CD14 | CI-966 | Not Approved | 1.591142 |
| ITGB2 | ERLIZUMAB | Not Approved | 1.458546 |
| ITGB2 | MLN-01 | Not Approved | 1.458546 |
| ITGB2 | BUTEIN | Not Approved | 1.458546 |
| ITGB2 | RECOMBINANT HUMAN THROMBOPOIETIN | Not Approved | 1.458546 |
| ITGB2 | MLNM-2201 | Not Approved | 1.458546 |
| CD14 | VB-201 | Not Approved | 1.193356 |
| CYBB | APIGENIN | Approved | 1.009763 |
| CYBB | LUTEOLIN | Not Approved | 0.820432 |
| ITGB2 | PANCREATIC PROTEOLYTIC ENZYMES | Not Approved | 0.729273 |
| ITGB2 | CYCLOOXYGENASE INHIBITOR | Not Approved | 0.729273 |
| ITGB2 | AME-133V | Not Approved | 0.729273 |
| ITGB2 | LIFITEGRAST | Approved | 0.729273 |
| ITGB2 | RECOMBINANT FAS LIGAND | Not Approved | 0.729273 |
| ITGB2 | VISILIZUMAB | Not Approved | 0.583419 |
| ITGB2 | MONOMETHYL FUMARATE | Approved | 0.583419 |
| CD14 | FLUTICASONE | Approved | 0.502466 |
| ITGB2 | ROVELIZUMAB | Not Approved | 0.486182 |
| ITGB2 | EFALIZUMAB | Approved | 0.486182 |
| ITGB2 | RECOMBINANT RANTES | Not Approved | 0.486182 |
| ITGB2 | RECOMBINANT LYMPHOKINE | Not Approved | 0.416728 |
| FCER1G | ASPIRIN | Approved | 0.372395 |
| ITGB2 | PHORBOL 12-MYRISTATE 13-ACETATE | Not Approved | 0.291709 |
| CD14 | LOVASTATIN | Approved | 0.244791 |
| ITGB2 | ENZYME INHIBITOR | Not Approved | 0.243091 |
| ITGB2 | SODIUM BUTYRATE | Not Approved | 0.208364 |
| ITGB2 | ANTIBIOTIC | Not Approved | 0.171594 |
| ITGB2 | H2O2 | Not Approved | 0.168294 |
| ITGB2 | METHYLPREDNISOLONE | Approved | 0.162061 |
| ITGB2 | HYDROGEN PEROXIDE | Approved | 0.145855 |
| ITGB2 | TETRADECANOYLPHORBOL ACETATE | Not Approved | 0.12683 |
| ITGB2 | SODIUM CHLORIDE | Approved | 0.104182 |
| ITGB2 | COLCHICINE | Approved | 0.084147 |
| ITGB2 | PENTOXIFYLLINE | Approved | 0.083346 |
| ITGB2 | THALIDOMIDE | Approved | 0.07884 |
| ITGB2 | INDOMETHACIN | Approved | 0.072927 |
| ITGB2 | PREDNISONE | Approved | 0.067839 |
| ITGB2 | CYCLOSPORINE | Approved | 0.050295 |
| ITGB2 | TRETINOIN | Approved | 0.048084 |
| ITGB2 | DEHYDRATED ALCOHOL | Approved | 0.036925 |
| ITGB2 | CYCLOPHOSPHAMIDE ANHYDROUS | Approved | 0.035574 |


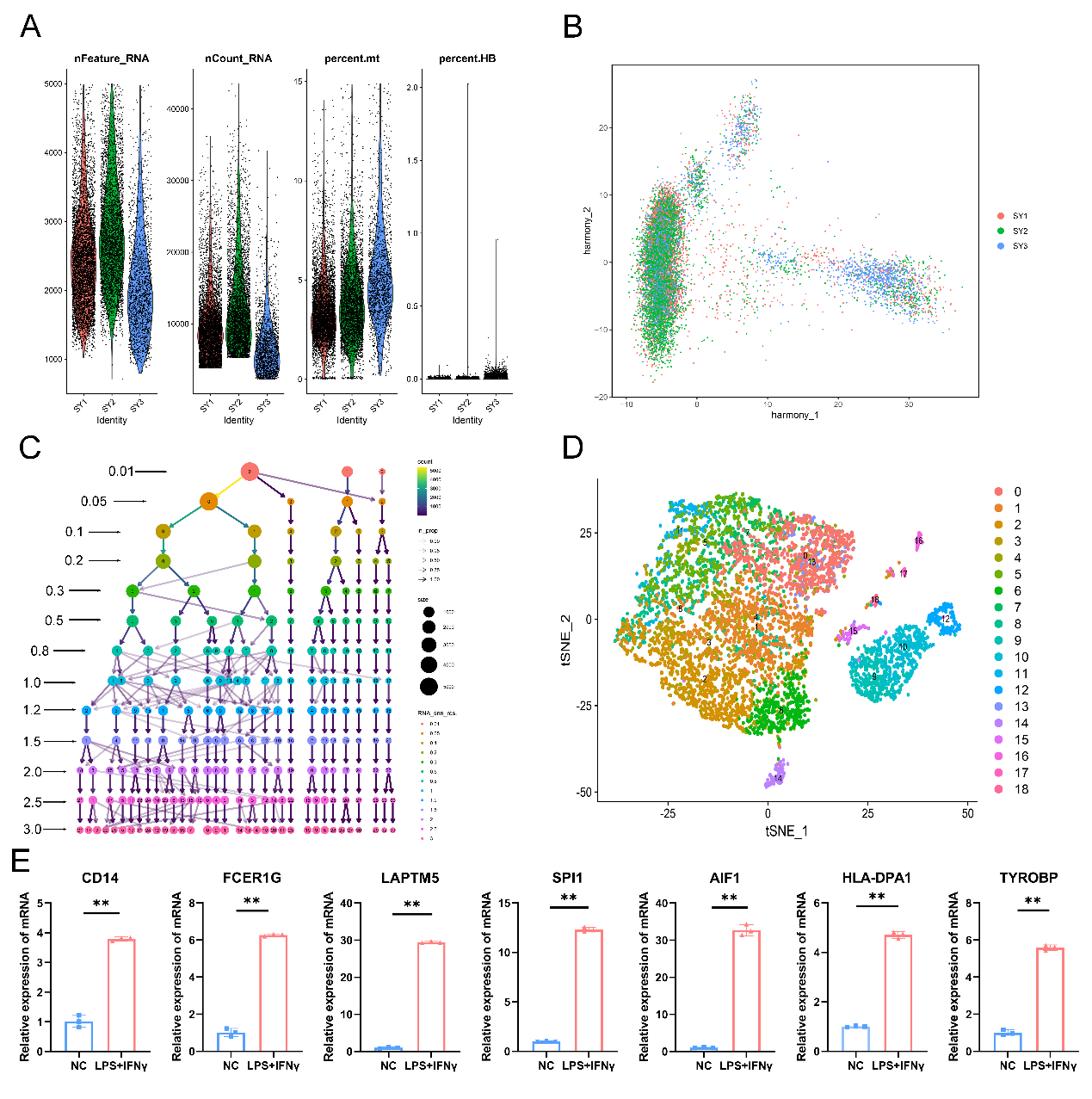


**Figure S1**. **Quality control of single-cell sequencing data and qPCR analysis of hub genes.**

(A) Violin plots showing the distribution of quality control. The plots display the number of features detected per cell (nFeature_RNA), the total counts per cell (nCount_RNA), the percentage of mitochondrial gene expression (percent.mt), and the percentage of hemoglobin gene expression (percent.HB). (B)Harmony-based dimensionality reduction plot, showing the distribution of cells from the three Synovial tissues (SY1, SY2, SY3) in the first two harmony components (harmony_1 and harmony_2). (C) Clustree plot showing the hierarchical relationships between the cell clusters. Each node represents a cluster, with the edges indicating the connections between clusters. The colors represent different cell types, and the size of the nodes reflects the relative proportions of cells in each cluster. (D) t-SNE plot showing the clustering of cells from all three synovial tissue. (E) mRNA expression levels of PYCARD-related key gene clusters in NC and LPS+IFNγ-stimulated cells.

**gh
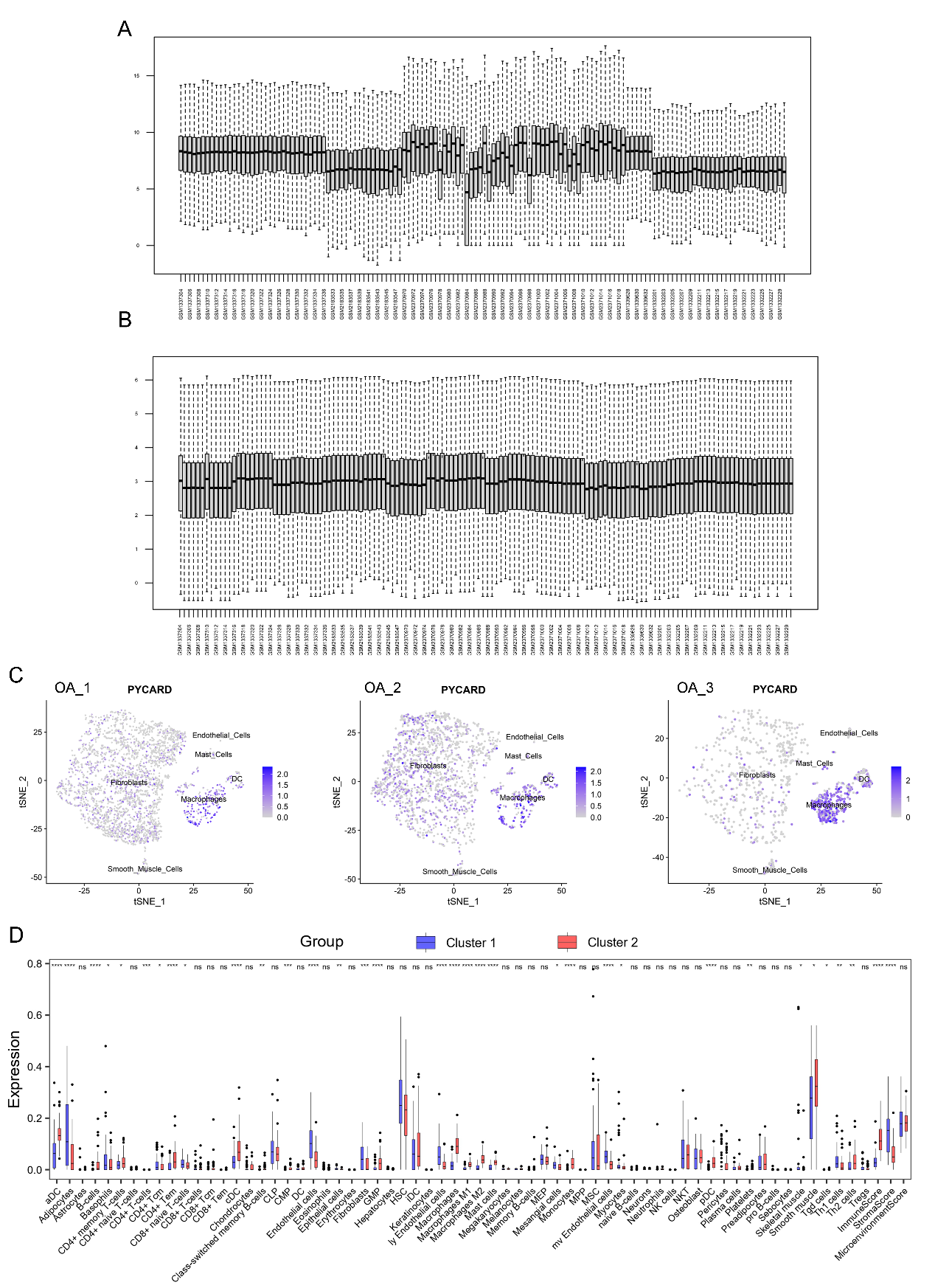
**

**Figure S2**. **Batch effect correction and visualization of immune infiltration.**

(A) Gene expression levels of each synovial sample before batch effect correction. (B) Gene expression levels of each synovial sample after batch effect correction. (C) Expression of PYCARD in 3 OA samples. (D) Box plot showing the expression levels of various immune cell populations between two clusters.
